# An evolutionary and structural characterization of mammalian protein complex organization

**DOI:** 10.1186/1471-2164-9-629

**Published:** 2008-12-23

**Authors:** Philip Wong, Sonja Althammer, Andrea Hildebrand, Andreas Kirschner, Philipp Pagel, Bernd Geissler, Pawel Smialowski, Florian Blöchl, Matthias Oesterheld, Thorsten Schmidt, Normann Strack, Fabian J Theis, Andreas Ruepp, Dmitrij Frishman

**Affiliations:** 1 Helmholtz Center Munich – German Research Center for Environmental Health (GmbH), Institute of Bioinformatics and Systems Biology, Ingolstädter Landstraße 1 D-85764 Neuherberg, Germany; 2 Department of Genome Oriented Bioinformatics, Technische Universität München, Wissenschaftzentrum Weihenstephan, 85350 Freising, Germany; 3 Max-Planck-Institute for Dynamics and Self-Organization, Bunsenstrasse 10, 37073 Göttingen, Germany

## Abstract

**Background:**

We have recently released a comprehensive, manually curated database of mammalian protein complexes called CORUM. Combining CORUM with other resources, we assembled a dataset of over 2700 mammalian complexes. The availability of a rich information resource allows us to search for organizational properties concerning these complexes.

**Results:**

As the complexity of a protein complex in terms of the number of unique subunits increases, we observed that the number of such complexes and the mean non-synonymous to synonymous substitution ratio of associated genes tend to decrease. Similarly, as the number of different complexes a given protein participates in increases, the number of such proteins and the substitution ratio of the associated gene also tends to decrease. These observations provide evidence relating natural selection and the organization of mammalian complexes. We also observed greater homogeneity in terms of predicted protein isoelectric points, secondary structure and substitution ratio in annotated versus randomly generated complexes. A large proportion of the protein content and interactions in the complexes could be predicted from known binary protein-protein and domain-domain interactions. In particular, we found that large proteins interact preferentially with much smaller proteins.

**Conclusion:**

We observed similar trends in yeast and other data. Our results support the existence of conserved relations associated with the mammalian protein complexes.

## Background

Knowledge of constraints governing systems provides a means to predict events and mechanisms that cause their breakdown. It may also allow one to speculate how such systems might have evolved. One class of biological systems that have captured much interest involves protein-protein interactions and protein complexes. Protein complexes are groups of two or more proteins that physically interact. Such interaction serves to spatially join, modify or create novel functional capability from component proteins. Once in a complex, proteins can achieve greater structural stabilization and protection from proteases, which result in significantly longer half-lives [1,2]. A breakdown in complex assembly has been associated with a number of diseases [3-5].

What forces have shaped the formation of complexes and what is their effect? The formation of quaternary structure is associated with greater constraint in the evolution of proteins [6-8]. Recently, a large collection of manually annotated mammalian complexes has become available in the CORUM database [9]. The combination of data from this resource and complexes derived from HPRD [10] and BIND [11] allows for one of the largest investigations of complex-specific protein constraints in mammalian species to date.

Recently, complexes have been investigated in the context of intrinsic disorder [12] and aggregation propensity of the subunits [13]. In this study, we focus on two characteristics of complexes: the complexity of a complex as represented by the number of unique proteins in a complex and the number of complexes a particular protein participates in. The number of protein subunits that bind together fluctuates in the context of different conditions. Complexes extracted from cells can be viewed as snapshot samples of proteins that have come together with adequate stability to be isolated. We subsequently derive 'complex participation' for individual proteins by counting the number of complexes that they belong to, in our large combined collection of protein complex samples.

Although these characteristics of complexes are dynamic, their relation to properties of component proteins derived in their isolated state can be studied. By deriving these relations, we hope to gain insight into certain constraints governing the organization of complexes. Here, we find evidence that protein length, predicted secondary structure and isoelectric point, as well as the nonsynonymous/synonymous substitution ratio of genes are associated with our measures of complex complexity and participation.

Available data on protein-protein interactions have been used extensively to predict complexes [14-25]. A strong discriminator between interacting and non-interacting protein pairs appears to be the presence of domains known to interact [26]. We assess the utility of known binary protein-protein and domain-domain interactions in predicting the composition and interactions in the mammalian complexes that we have collected.

## Methods

### Complex and Interaction data

1732 experimentally verified protein complexes annotated at MIPS [27] and stored in the CORUM database [9] were combined with 631 complexes retrieved from HPRD [10] and 538 complexes from BIND [11]. After removal of redundancy, there were 2706 different complexes containing 4543 different proteins from human, mouse, rat, dog, rabbit, cow, pig and other mammals. Of these 2706 unique complexes, 665 are subcomplexes of other complexes in the data. A more detailed description of the complexes appears in the additional files [see Additional file 1].

The focus of this study was on mammalian complexes but we also analyzed the MIPS yeast complexes stored in CYGD [28]. We use a set of 1142 unique yeast complexes consisting of 2755 proteins. A much larger set of yeast complexes consisting of 5370 proteins in 2025 complexes was produced by merging the MIPS yeast complexes with the 893 complexes predicted by Friedel et al. [25]. We will refer to this set of complexes as the 'extended yeast complex set'. Possible estimates of the completeness of the human and yeast complex data appear in the additional files [see Additional file 1].

We assembled 94906 pairwise eukaryotic protein-protein interactions from BIND, DIP [29], HPRD, MINT [30], Mpact [31] and MPPI [32]. 29074 of those interactions were between different proteins from human, mouse or rat and were used in this study. We also retrieved 127514 binary protein interactions by over 36500 proteins from IntAct [33]. We will refer to interaction data from IntAct in the text specifically with the word 'IntAct' to distinguish from the interaction set we collected.

We refer as domain-domain interactions in the text as those derived from known 3D structures of proteins, obtained from the iPfam [34] and 3did [35] database. There are 3654 such interactions between protein domains in total. Pfam domains of protein sequences were taken from Uniprot [36]. We assume that peptide chains in PDB structures with C-beta atoms within 8 Angstroms of each other interact.

We generated random complexes using two different random models to evaluate significance of trends associated with non-stochastic protein complex organization. For the random model in the main text (Model 1), we generated the same number of random complexes as found in annotated complexes by picking the same number of proteins as found in each of the annotated complexes from a pool of all proteins collected from these complexes. We picked proteins randomly with replacement throughout the process of random complex generation to simulate neutrality of protein reuse in the complexes. A second random model (Model 2) of the complexes which uses the complex complexity and participation preserving rewiring method, first introduced by Maslov and Sneppen [37] was also tested [see Additional file 1]. In certain parts of this paper, we used sets of randomly generated complexes (each set containing the same number of complexes as found in the annotated complexes) in Monte-Carlo simulations (denoted MC-test) to derive p-values for significance statements. P-values of events were estimated by the fraction of random simulations supporting the null hypothesis.

### Computed features of genes and proteins

Secondary structure was predicted with PSIPRED [38]. Our conclusions did not change when we repeated our experiments using SSPRO [39]. The performance of PSIPRED in terms of secondary structure content prediction is benchmarked [see Additional file 2]. We measured human protein evolutionary rates by non-synonymous to synonymous substitution (*dN/dS*) ratios computed from coding sequences of ortholog pairs from Ensembl [40]. Yeast (*S. cerevisiae*) gene *dN/dS *ratios were computed using orthologs [41] from *S. mikatae *by PAL2NAL [42] with default parameters and Codeml from the PAML package [43]. The smallest *dN/dS *ratio was recorded for each gene when more than one potential ortholog was identified (our conclusions did not change if we chose the larger one). The values of *dN/dS *depend on the calculation methodology but they should be comparable within the same species pairs chosen. Note that only *dN/dS *ratios less than 1 (computed from human-mouse orthologs) were associated with the annotated human complexes. The mean *dN/dS *ratio of individual genes encoding a given protein complex was taken as a measure of selection associated with the protein complex. We focused on a measure of evolutionary rate reflecting more on changes in protein sequence. We considered evolution at synonymous sites [44,45] to be neutral.

We refer to "pI" in the text as values obtained from BioPerl-based [46] isoelectric point prediction on protein sequences. Essential genes for *S. cerevisiae *were taken from CYGD [28]. Essential genes for human were gathered by merging those from [47] and Table S6 of [48].

### Statistics of trends in 2-D plots

We assessed trends concerning 2-dimensional protein complex data by first applying standard linear regression to see if there is a noticeable slope different from a horizontal line (observed when the y values do not depend on the x values). We considered a given trend to be significantly associated with non-stochastic protein complex organization if the p-value reported by the t-test on the slope of the regression line (the null hypothesis being a slope = 0) was less than the maximum value observed when 1000 sets of random human or yeast complexes were generated. For example, when random complexes were generated from entire sets of annotated complexes, p-values associated with plots between mean *dN/dS *values and complex complexity for these random complexes were always above 0.003; so we set this value as a threshold of significance for such trends. However, the use of the t-test by itself is potentially inadequate because it could be significantly affected by biases in our snap-shot protein complex samples. So in addition, we repeated the same plots using sets of complexes likely to have different biases. These include the MIPS yeast complexes, the extended yeast complex set, sets of complexes where subcomplexes were removed and sets of complexes in different subcellular compartments. Unless otherwise stated, only those trends in which all assessments (from the regression line analysis to the analysis with other complex datasets) are considered significant are reported. Analysis was aided by the PROMPT tool [49].

## Results and discussion

### Complex Complexity and Participation

**Figure 1:**
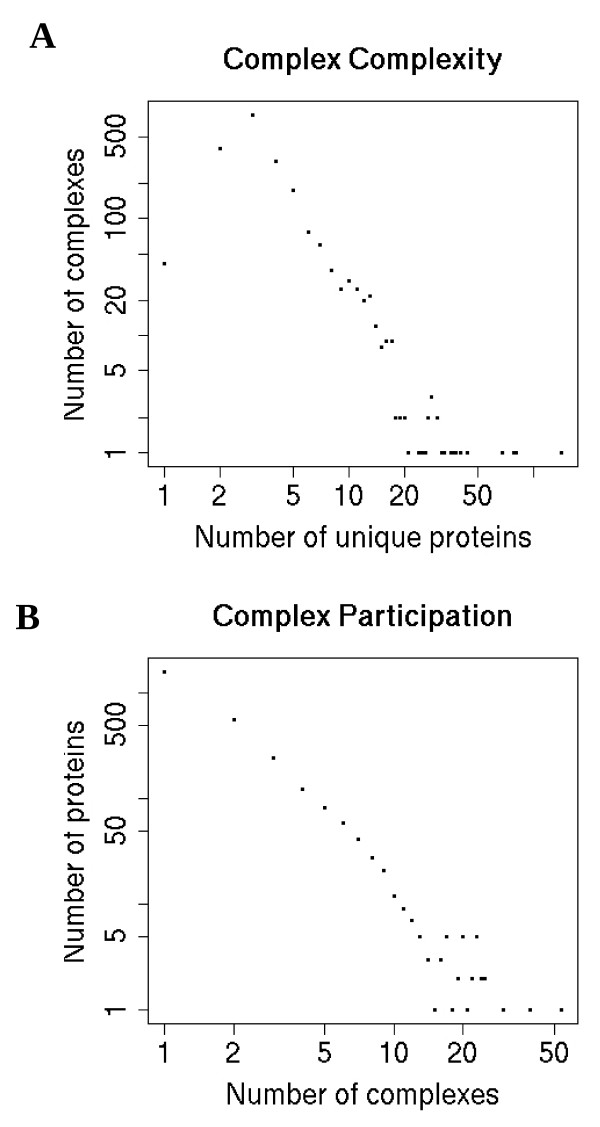
**Composition of mammalian complexes**. A) The number of complexes (y-axis) with a particular number of unique proteins (x-axis) is plotted. B) The number of proteins (y-axis) participating in a particular number of complexes (x-axis) is plotted.

We first examined the distribution of complex complexity, the number of unique proteins in a complex. Amongst all mammalian complexes collected, the number of unique proteins ranges from one for homo-oligomers to 142 (U2-type spliceosome [CORUM:351]). When the number of unique proteins increases past 4, complexes become increasingly rare following a power law-like distribution (Figure 1A). When we considered yeast complexes, we saw similar trends [see Additional file 3].

Not all proteins are exclusive to a single complex. Having defined complex participation as the number of complexes that a particular protein is found in, we find that it also follows a power law-like distribution (Figure 1B, [Additional file 3]). Some of the proteins currently with the highest complex participation include human integrin beta-1 [Uniprot:P05556], histone deacetylase 1 [Uniprot:Q13547] and RING-box protein 1 [Uniprot:P62877] which were annotated to be in 54, 39 and 24 complexes, respectively so far.

The decrease in the number of complexes as the number of subunits increase might be a reflection of the increased difficulty in assembling and thus evolving larger beneficial complexes. Although this trend seems to follow a power law, as commonly reported for biological networks [50], we could not ascertain if this truly is the case. Similarly, we could not ascertain whether complex participation follows a power law. Our observed complex complexity and participation distributions may be the result of our complexes being samples of mammalian proteins [51,52] derived under a variety of conditions [Additional file 1]. The number of unique proteins in yeast complexes was also observed to follow a similar-shaped distribution and an exponential model of the data has been proposed [53,54]. In both mammalian and yeast complexes, most proteins belong to few complexes, thus supporting the idea of modularity [55] between complexes in the context of the protein-protein interaction network that have been examined.

### The length of proteins in complexes

**Figure 2:**
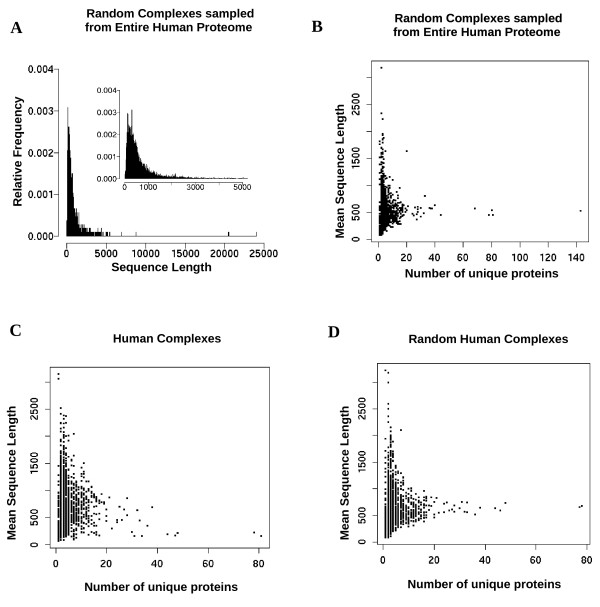
**Protein length in human complexes**. A) Length distribution of proteins in human complexes generated randomly from the entire known human proteome. Mean lengths of proteins versus the number of unique subunits in: B) model 1 random complexes generated from the entire proteome C) annotated human complexes D) model 1 random human complexes generated from data we collected.

One of the simplest protein properties to study is the length of the protein in terms of the number of amino acids. The distribution of human protein lengths is skewed towards smaller values and subsequently when we sample uniformly from this distribution to generate random complexes, the distribution of the protein lengths remains skewed (Figure 2A). The mean length of proteins in this distribution is ~500 amino acids (Figure 2A).

By picking proteins with replacement from the distribution of proteins in Figure 2A, we generated random complexes and plotted the complexity of the complexes in terms of the number of unique subunits against the mean length of these subunits (Figure 2B). As complex complexity increases the upper bound for mean lengths of proteins drops (before 20 unique proteins) and levels out at ~510 amino acids for more complex complexes. Because the mean length of proteins in the human proteome which we sample from is ~500 amino acids, as one adds more random proteins to complexes, the mean length of proteins in the complexes tends to stabilize near 500 amino acids. This observation was similarly made in annotated human complexes (Figure 2C) and random complexes generated from this annotated data (Figure 2D). A similar asymptotic bound is thus formed in all our plots. For example, mean lengths of 2000 amino acids were absent in complexes of more than 20 subunits in both the random and the complexes that we have collected. Like for the random human complexes, the mean protein length distribution for the real human complexes (Figure 2C) was also skewed towards smaller protein lengths. Similar plots were observed for all mammalian complexes that we collected and Model 2 random complexes [Additional file 4]. These results suggest that the mean protein length in complexes reflects to an observable degree the length distribution of proteins encoded in the proteome (Figure 2A). Similar observations were made when the median length of proteins was studied in the context of complex complexity [Additional file 5].

In contrast to randomly generated complexes, the complexes that we have collected had much more variable mean lengths, especially for complexes with large numbers of subunits. We observe this as an increased scattering of points on the right side of the plot (Figure 2C). This phenomenon was similarly observed in yeast complexes [Additional file 6]. In particular, when complex complexity increases past 20 different subunits, complexes with mean protein lengths below 250 amino acids are rarely generated randomly (Random Model 1: P < 10^-6^) but are present in annotated mammalian and yeast complexes. For example, the 81 subunits of the human ribosomal complex [CORUM:306] have a mean length of 169 amino acids and the 37 subunits of the mouse mitochondrial NADH dehydrogenase complex [CORUM:381] have a mean length of 234 amino acids.

Although the complexes analyzed are samples of what is in cells, it is a large set that has been manually annotated. Assuming that the plots in Figure 2 capture length constraint bounds, they can provide rules of thumb for spot-checking errors in newly isolated complexes. For example, if an isolated human complex of 10 subunits had a mean subunit length of 2000 amino acids, our plots suggest that large numbers of additional missing subunits of length 2000 or more are unlikely. If such a complex did exist, then it would be striking given both the annotated complex data (Figure 2C) and data generated from random complexes (Figure 2B, Figure 2D; [Additional file 4]). Unusual property distributions associated with complexes provide initial suggestions of errors in the composition of the complexes or unusual constraints associated with these complexes. For example, the average length of a large sample of human mitochondrial proteins appears to be much smaller than the average length of all human proteins (means ± standard deviation/medians are: ~320 ± 304/235aa vs. ~500 ± 551/364aa, respectively; MW, KS-test (Mann-Whitney, Kolmogorov-Smirnov tests): P < 10^-57^) in the Eukaryotic Subcellular Localization Database [56] [Additional file 7]. Similar results were obtained when we used the reference set from MitoP2 [57] as a source of mitochondrial proteins. Despite large amounts of protein turnover since the mitochondria prokaryotic origin [58], most (> 97%) human mitochondrial proteins have lengths less than 1000 amino acids. These results suggest unusual size constraints associated with mitochondrial proteins. Mitochondria-related functions, the need for proteins to import into the mitochondria [59], the presence of reactive oxygen species which can impose difficulties for protein folding [60] are possible reasons which may have helped limit the incorporation of very large proteins into mitochondria. The scarcity of large proteins in the mitochondria, in turn, will likely limit mean protein lengths of its complexes. Although many (300/1962 = 15%) human complexes in our data have mean protein lengths over 1000 amino acids (Figure 2C), none of the 28 mitochondrial human complexes we annotated so far exceed this limit. If mean protein lengths in complexes reflect protein length distributions, we expect hetero-oligomeric mitochondria human complexes with mean protein lengths over 1000 amino acids to be extremely rare.

The relative lack of complexes with many (>20) different large proteins (> 1500aa) in both mammals and yeast suggests that evolving such complexes is also extremely difficult. Smaller subunits may be easier to fold, transport [61], require less conformation sampling [2] and undergo smaller entropic loss [[62] – Supplement] during complex assembly. These factors may have contributed to the absence of highly complex complexes with many large proteins in our data.

### Natural selection and complexes

**Figure 3:**
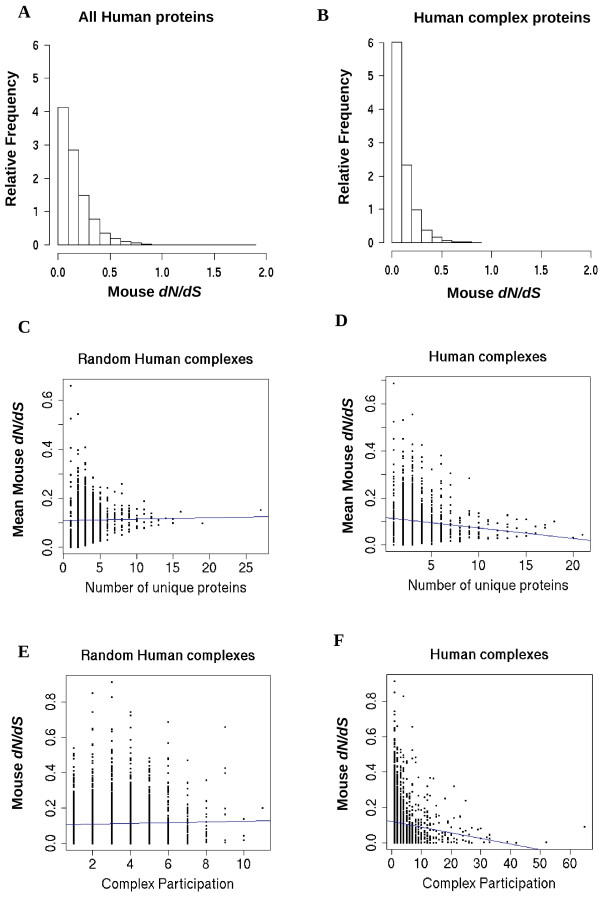
**Complexes and selection**. *dN/dS *distribution of genes associated with A) all human proteins and B) proteins in the human complex data. D) The mean *dN/dS *ratio for human-mouse orthologs is plotted against the number of unique proteins in the complex. Complexes with more unique proteins have a significantly smaller mean *dN/dS *ratios than those with less unique proteins (t-test: P < 3.1 × 10^-7^). F) The *dN/dS *ratio for human-mouse orthologs is plotted against the number of complexes they participate in. Proteins participating in more complexes have significantly lower *dN/dS *ratios than those with less complex participation (t-test: P < 7.4 × 10^-9^). C, E) Both trends are rarely observed for model 1 random complexes.

To understand observations from a more evolutionary point of view, we analyzed non-synonymous to synonymous substitution ratios (*dN/dS*) derived from human coding sequences and their alignments with orthologs from mouse (Figure 3). One way of comparing selection on coding sequences associated with different complexes is by their mean *dN/dS *ratios derived from the same species pairs.

For example, proteins from the human MBD1-MCAF complex [CORUM:2759] are encoded by two genes with a mean *dN/dS *ratio of 0.37, computed from mouse orthologs. The sequences coding for this complex seem to be under much less selective constraint than ones coding the eIF3 complex [CORUM:742] which is associated with a mean *dN/dS *ratio of 0.04 (Stdv. = 0.03, median = 0.02).

It has been noted previously that easily alignable eukaryotic mRNAs are over-represented for multi-subunit complexes [63] due to increased sequence conservation. In terms of trends, we find that as the complexity of the complex increases, the mean *dN/dS *ratio of human genes of associated constituent proteins tends to decrease suggesting that complex complexity negatively correlates with purifying selective pressure on genes (Figure 3D). The trend is not extremely strong but such a trend was not observed with random complexes (Figure 3C; [Additional file 8]). In particular, complexes with more than 15 proteins and mean *dN/dS *ratios below 0.07 are absent in our random complex set. For example, the 20 genes from the PA700 proteosome regulator complex [CORUM:32] have a mean *dN/dS *ratio of 0.03 but such complexes are not likely generated randomly (Random model 1: P < 0.05). A contributing reason can be derived by examining the distribution of *dN/dS *ratios. The distribution of human-mouse *dN/dS *ratios is skewed towards small values considering all known human genes with over 40% of the values < 0.1 (Figure 3A). The distribution of *dN/dS *ratios associated with our sample of annotated complexes (Figure 3B) is likewise skewed with 60% of the values < 0.1. In both cases, the mean *dN/dS *ratio is close to ~0.1. As more randomly-selected proteins are added to form random complexes, the mean *dN/dS *ratio for genes associated with the complexes tends to stabilize also at 0.1. Thus, highly complex complexes with mean *dN/dS *ratios substantially below 0.1 are rarely generated randomly.

Knowing that many proteins belong to more than one complex, we also asked how this fact was related to selective pressure. We find that as the number of complexes a protein participates in increases, the *dN/dS *ratio of corresponding genes also tends to decrease (Figure 3F), suggesting that complex participation also correlates negatively with the *dN/dS *ratio. This trend is not observed when complexes were generated randomly by picking proteins with replacement (Figure 3E). From such randomly generated complexes, proteins participating in more than 10 complexes rarely occur, but they are clearly present in our annotated data. Binned average plots of Figures 3D and 3F appear in the additional files [see Additional file 9].

Because such results could potentially be sensitive to the ortholog pairs compared, the quality of the gene models, and peculiarities of human/mouse evolution, we also repeated such analyses using a variety of other mammals [see Additional file 10]. For human-dog, human-chimp and human-rat orthologs, we observed similar trends although in the closely related human-chimp case in which complex complexity was plotted against mean *dN/dS*, the trend was not statistically significant. We also had similar observations with yeast complexes using *S. cerevisiae *– *S. mikatae*, *S. cerevisiae *– *S. paradoxus *orthologs. We did not observe such trends from *dN/dS *ratios derived from the more distant pairing of *S. cerevisiae *– *S. castellii *orthologs (data not shown). For both mammalian and yeast complexes, the trends appear to be conserved using *dN/dS *ratios generated from a variety of ortholog-pairs with some exceptions.

Related to *dN/dS *ratios is the gene conservation of orthologs of the proteins in complexes. Compared to *dN/dS *ratios, statistically significant differences in gene conservation are even more difficult to detect in closely related genomes. However, over large evolutionary distances we noticed that highly complex complexes tend to have conserved their subunits. For example, between yeast and human, yeast complexes with more than 60 subunits had more than 80% of their subunits also encoded in human. This was not observed in random complexes [Additional file 11]. Yeast complex proteins conserved in human also participated in a significantly higher number of complexes than those without a human ortholog (mean ± standard deviation/median complex participation of 8.0 ± 6.8/6.0 vs. 4.2 ± 4.3/3.0; MW, KS-test: P < 10^-70^). A significant difference in complex participation was not observed for model 1 randomly generated complexes. These results concerning *dN/dS *ratios and gene conservation portrays similar relationships between gene evolution, complex complexity and participation at different time and constraint scales.

Because our conclusions are based on a limited sample of complexes, we sought for extra datasets of protein complexes. The combination of the annotated MIPS yeast complexes with those that were predicted [25] allowed us to test our hypotheses on a large set of complexes covering 5370 (88%) of the proteins encoded in the yeast proteome. We continued to observe a significant negative correlation between mean *dN/dS *with complex complexity and *dN/dS *with complex participation using such an extended complex dataset [Additional file 12].

Often in a cell, complexes exist along side their subcomplexes. In our data, subcomplexes of complexes are present. If we repeated our analysis with subcomplexes removed, we still observed a significant negative correlation between mean *dN/dS *with complex complexity and *dN/dS *with complex participation [Additional file 12]. Similar trends were also observed when we considered nuclear localized and non-localized complexes separately [Additional file 12].

As complexes are assembled or disassembled, the mean *dN/dS *ratio should fluctuate as proteins are added or subtracted from the complex. Nevertheless, there also seems to be an asymptotic bound for mean *dN/dS *ratios as complex complexity increases and *dN/dS *ratios as complex participation increases. The conserved trends we observe may be a reflection of these bounds. We find that genes associated with our human complexes which are considered essential (see Methods) tend to have lower *dN/dS *ratios (computed by human-mouse orthologs) and encode proteins that participate in more complexes (MW, KS-test: P < 0.01) compared with all genes in the human complexes. We see the same significant trend for yeast (*dN/dS *ratios computed with *S. cerevisiae *– *S. paradoxus *orthologs). These results suggest that for proteins participating in increasing number of complexes, lower *dN/dS *ratios tend to be associated with purifying selection to maintain organism fitness. Essential *S. cerevisiae *complex proteins tend to be found in complexes with more unique subunits (MW, KS-test: P < 0.05) compared to other proteins in the yeast complex data. However, such a trend was not observed for human possibly because a significant number of proteins which contribute to complex complexity are not known to be essential. We conclude that there is strong evidence of non-random association between *dN/dS *ratios of genes and complexes of their protein products.

The finding that the number of protein-protein interactions positively correlates with the level of conservation of orthologs between species [64,65] is similar to our finding that highly complex complexes tend to conserve their orthologs. Previously, increased gene conservation has been correlated with lower evolutionary rate [66]. We have found that highly complex complexes tend to conserve their orthologs and have lower *dN/dS *ratios, in agreement with these results.

In summary, we observed that the mean *dN/dS *ratios of proteins in the collected complexes tend to decrease with increasing complex complexity. There seem to be at least two major phenomena associated with this observation:

1) The skewed distribution of *dN/dS *ratios amongst genes as depicted in Figure 3B. In particular, as complex complexity increases, the mean *dN/dS *ratios of proteins in complexes tend to stabilize towards the mean of this skewed distribution.

2) increased selective pressure on proteins involved in the organization and function of more complex complexes

The median of the mouse *dN/dS *values (Figure 3B) is 0.08 which is less than the mean of 0.1. When we plotted Figure 3D and related supplements using median *dN/dS *values instead of the mean, we also found the median *dN/dS *decreased significantly with increasing number of unique subunits. Such a trend was consistently found when human-mouse, human-dog, human-rat, *S. cerevisiae *– *S. paradoxus *and *S. cerevisiae *– *S. mikatae dN/dS *median values were used (for all plots, t-test: P < 7.1 × 10^-7^; selected plots are shown in the additional files [Additional file 13]).

Thus, for the complex data available, we observed that both the mean and median *dN/dS *ratios of genes negatively correlates with the complex complexity.

### Homogeneity of Protein and Gene Properties

Next, we examined similarities between proteins within mammalian protein complexes (Table 1). Although not necessarily similar to physiological pI, our predicted pI values reflect sequence properties which may be of some utility. We ask whether subunits in complexes tend to have greater homogeneity than expected in terms of predicted pI values. Considering complexes of 3 or more unique proteins, we found that the standard deviations in protein pI are significantly lower in the annotated complexes compared to those in model 1 random complexes. Thus, the pI of a protein can be a feature which may be useful in predicting whether the protein belongs to a particular complex or not. The need for subunits to co-localize [67] and the presence of highly sequence similar paralogs in complexes [68] should be contributing factors to our observation of lower than expected pI deviation between complex proteins.

**Table 1:** Homogeneity of protein properties in mammalian complexes of 3 or more proteins.

Protein Complex Data	Property	Mean Stdev. Annotated	Mean Stdev. Random
All mammalian	pI	1.5 ± 0.7 (1.5)	1.8 ± 0.7 (1.8)
All mammalian	% Helix	14.4 ± 7.8 (13.5)	18.6 ± 7.5 (18.6)
All mammalian	% Sheet	9.5 ± 5.9 (8.7)	10.9 ± 5.4 (10.3)
All mammalian	% Coil	10.3 ± 5.0 (9.8)	13.3 ± 5.6 (13)
Human (against mouse)	*dN/dS*	0.07 ± 0.05 (0.06)	0.09 ± 0.06 (0.08)

The standard deviations of protein property values for each annotated complex are significantly smaller than ones for model 1 random complexes (MC-test using 1000 complexes generated randomly with replacement: P < 0.001). The means ± standard deviation of these standard deviations for each property are shown in the last two columns along with median values (in parentheses).

The common presence of paralogs in complexes and the correlation between secondary structure and localization [69] also contributes to explaining why we observed significantly lower than expected deviations of secondary structure content (percent helix, sheet and coil) in proteins belonging to the annotated complexes versus those in random complexes.

Similar to protein properties, we also found that the *dN/dS *ratio of genes associated with annotated human complexes are significantly more homogeneous compared with those from randomly generated complexes. Some examples of complexes with very low deviation of *dN/dS *values include Ubiquitin E3 ligase [CORUM:386] and the MKK4-ARRB2-ASK1 complex [CORUM:1297] with a standard deviation of 0.0007 and 0.001, respectively. The complex with the highest observed *dN/dS *deviation of 0.34 is the DNA ligase IV-XRCC4-XLF complex [CORUM:359]. Significant differences between random and annotated complexes were also observed when the *dN/dS *ratio was computed with dog, rat, or chimp orthologs (data not shown). These observations agree with previous results providing evidence that yeast proteins belonging to the same functional module tend to have more similar evolutionary rates than those belonging to different modules [70]. We observed similar results when we examined yeast complexes [Additional file 14] and when we generated model 2 random mammalian and yeast complexes (data not shown).

To conclude, we suggest that pI, secondary structure and *dN/dS *ratios can be used to help predict the probability that a protein belongs to a particular complex. We did not observe a significant difference in standard deviations of protein length between proteins belonging to annotated versus Model 1 random mammalian complexes. It may still prove useful for the separation between real and erroneous complexes when combined with other properties.

### Comparison with pairwise protein interactions

**Figure 4:**
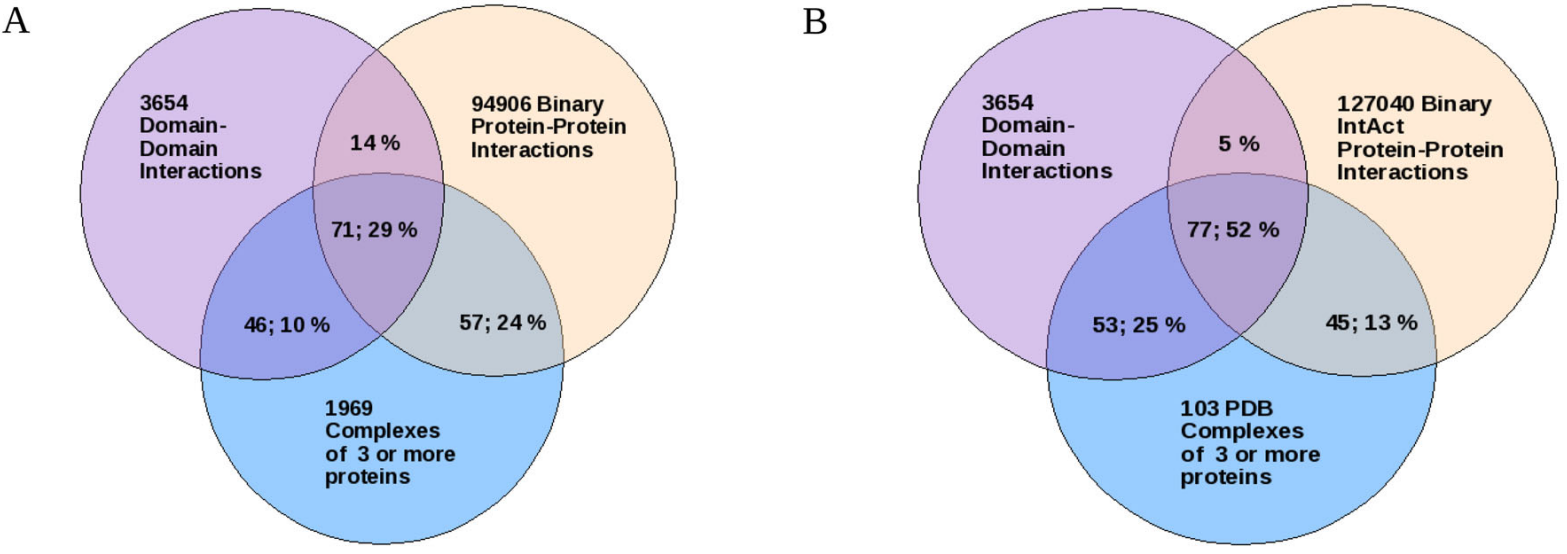
**Coverage by binary domain and protein interactions**. Coverage is indicated by values in the Venn diagrams. Example: A) Protein content coverage. 71% of the proteins in our complexes with 3 or more unique proteins can be explained by either domain-domain or binary protein interactions. The full protein content of 29% of these complexes can be explained by either interaction-type. 14% of binary ppi can be explained by predicted domain-domain interactions. B) Interaction coverage. 5% of IntAct binary interactions could be explained from predicted domain-domain interactions. 77% of solved protein-protein contacts in our complexes of known structure with 3 or more unique proteins can be explained by either domain-domain or binary protein interactions. All solved contacts in 52% of these complexes can likewise be explained by these interactions.

The overlap between binary protein-protein and predicted domain-domain interactions with proteins in our complexes was assessed (Figure 4). On average, 57%, 46% and 71% of the proteins found in the complexes (of 3 or more proteins) can be explained by known binary protein-protein, predicted domain-domain or either type of interactions (see Methods), respectively. The entire annotated protein complement of 24%, 10%, and 29% of these complexes can be explained by binary, domain-domain or either type of interactions, respectively. In addition we find that 14% of binary protein-protein interactions can be explained by domain-domain interactions which is in the range of 4–19% identified by Schuster-Bockler & Bateman [71].

192 PDB structures are available for our complexes (103 of which consist of at least 3 different proteins). A large proportion of these structures are solved with protein fragments rather than the full-length proteins. Many of the interactions between peptides in these structures (see Methods) are supported by binary protein-protein interactions. The IntAct interaction set (excluding interactions derived only from NMR or X-ray diffraction) covered on average ~40% of detected interactions in a given complex containing PDB structure. Interactions in 34 (18%) PDB structures were fully explained by these IntAct binary interactions. A similar coverage (Figure 4B: 45% mean interaction coverage; 13% of complexes with all interactions fully explained) is provided by these IntAct binary interactions even if we consider only complexes with 3 or more distinct interacting peptides. In contrast, on average 53% of interactions in complexes with 3 or more distinct interacting proteins are explained by predicted domain-domain interactions. Predicted domain-domain interactions explain all interactions in about 25% of such complexes. Our results suggest that known protein-protein and domain-domain interactions can aid substantially in predicting interactions in our complex data. Many of these binary and domain-domain interaction defined protein pairs may be subcomplex precursors of the mammalian complexes which form during the course of assembly. Some of these subcomplex precursors might be functional complexes in current mammalian and ancestral organisms [62].

**Figure 5:**
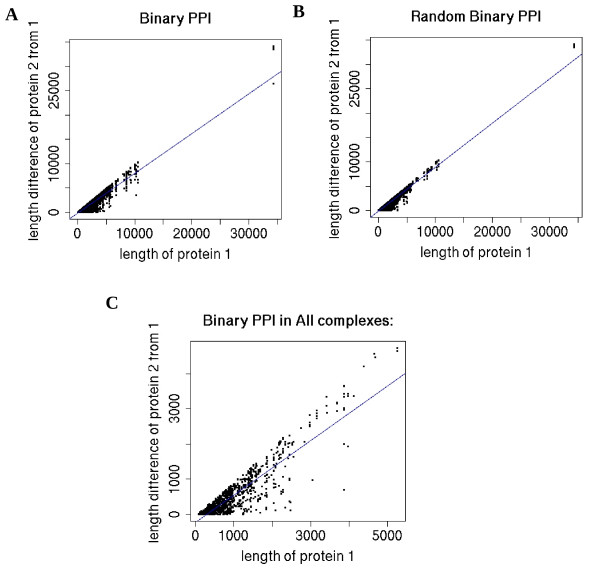
**Large proteins tend to interact with relatively smaller partners**. For each protein pair of different proteins, the length of the larger protein (protein1) is plotted on the x-axis. A,B,C) The difference in length with its partner (protein2) is plotted on y-axis. Larger proteins tend to have a larger length difference with their partners compared to proteins of smaller size. We see this trend amongst A) IntAct binary protein-protein interactions and B) Random binary interactions generated from the IntAct data C) binary interactions mapped onto all collected mammalian complexes. For all plots A-C, the trends are significant (t-test: P < 0.001).

Considering binary protein-protein interaction pairs in IntAct, those found in our complexes, and those derived from contacts in PDB structures associated with our mammalian complexes, we found that larger proteins tend to have larger length differences with their interacting partners (Figure 5, [Additional file 15]). For example, a large protein of length 5000 amino acids was more likely to be found interacting with a protein of ~1000 amino acids (a 4000 amino acid difference) rather than another protein of 5000 amino acids. However, a protein of length 300 amino acids often was found interacting with another protein of similar size. A number of reasons can explain this observation. First, the length distribution of the proteins is skewed such that relatively long proteins tend to be rare (Figure 2A). Second, on average shorter genes appear to be more abundantly expressed [72,73] than longer genes. Thus, relatively large proteins may have a greater chance to encounter shorter proteins rather than longer proteins. The observation that many complex complexes tend to be composed of proteins with a shorter mean length may also be related to this phenomenon since the evolution of large complexes may depend on how often and easy it is to bring together undamaged components. The relatively low *dN/dS *ratios of genes from highly complex complexes may also be associated with this phenomenon (Figure 3D). Interestingly, similar observations (fitting even better to the straight line) were made with random protein-protein interactions (generated by picking proteins in each interaction pair randomly with replacement) from the IntAct data (Figure 5B). The relative low abundance of relatively large proteins in both the real and random interaction data can explain the trends in Figure 5A–C. The differences between real (Figure 5A, C) and random (Figure 5B) plots might be attributed to historical, selective constraints or experimental error on certain protein-protein interactions. Binned plots of Figure 5A are found in [Additional file 15].

### Explaining evolutionary rate

In this work, we have hinted at various explanations such as the ease of complex assembly to reason why more complex complexity and participation negatively correlates with our measures of evolutionary rate. We believe that these explanations are contributing factors but clearly a systems approach [74] is warranted. The ability of a protein to change sequence is related to the mutability of its encoding gene, its subsequent expression, the consequences of errors in the gene's products which is related to the magnitude of expression and finally the degradation of those products. Fitness effects for the individual and the population under historical environmental constraints needs to be taken into account to completely understand why certain sequences have been transmitted for millions of years of evolution. A challenge is to uncover which factors are more important than others in explaining evolutionary rate [75-77]. Factors associated with proteins early in their expression such as transcription and translation [78] error rate or more general factors such as protein abundance would affect the evolutionary rate of all proteins. However, factors which are related to later events in a protein's life cycle such as functional molecular interactions will likely explain evolutionary rate differently for different types of proteins. For example, transcription factors which bind DNA will have sequences constrained by the need to bind DNA. Such constraints would be absent for those proteins that do not bind DNA. If shorter proteins are more abundantly expressed, and more complex complexes tend to contain smaller proteins, higher expression of smaller proteins in more complex complexes could help explain our observations of lower dN/dS ratios. While expression appears to be important, the functional and non-functional [79] interactions of the protein and its precursors with other proteins [7,80] or molecules most likely play a major role in defining the protein's relation to organism fitness. It can be very challenging relating evolutionary rate to these different factors, especially when some of these factors are measured with substantial noise or are unknown [81,82]. While the processes leading up to complex assembly can help explain protein evolutionary rate, there may be factors not directly related to complex formation yet to be fully accounted for. Some progress has been made in teasing apart contributions to evolutionary rate [83].

Part of the reason why deriving mechanisms explaining evolutionary rate has captured so much interest is because such data is readily available from comparative genomics. However, without knowing mechanisms explaining evolutionary rate values, its significance and the utility of associated information becomes limited. In particular, its relation to protein function and disease needs to be worked out. By combining other information such as those derived from complexes, one might gain a deeper insight. For example, just knowing whether a protein complex contains a certain number of proteins or whether a protein belongs to a certain number of complexes may significantly help us predict its relation to certain fitness effects and future diseases, given information on its evolutionary rate. It has also been observed by many that proteins related to genetic diseases in OMIM [84] tend to be relatively long [85,86] and it might be interesting to work out how this relates to specific sequences and structural features in the complexes.

## Conclusion

In conclusion, we have found relations and trends between the *dN/dS *ratio, primary sequence properties, secondary structure, complex complexity, participation and localization using large samples of protein complexes derived under certain distributions of conditions. There is a large evolutionary distance between yeast and mammals. Protein complexes and their subunits are not necessarily conserved between distant species [87]. The different environments which mammals and yeast occupy may have produced different evolutionary pressure on the protein complexes. Despite these differences, many of our observations appear to be conserved for the mammalian and yeast complex data that we have. We suggest that our observations have been significantly influenced by constraints during the evolution of the complexes. Because of these constraints, numerical boundaries were discovered when we related properties such as protein length to complex complexity. Proteins at these property value boundaries are interesting because they are exceptional and possibly point to unusual pressures or homeostatic adaptations that allow for their presence in cells. For example, by studying highly complex protein complexes composed of very large proteins, one might uncover new mechanisms that allow for their assembly and the assembly of later evolved proteins. We also observed that random phenomena (as in neutral drift) from skewed origins can appear highly stable when averaged over large numbers (Ex. Figure 2B, D; Figure 5B). Compared to randomly generated phenomena, biologically-derived characteristics can appear less structured despite these circumstances (Ex. Figure 2C, Figure 5A). Our focus in this study is mainly on information about the presence of certain protein complexes. Once data on the abundance, stoichiometry and dynamics of mammalian protein and protein complexes become available on a large scale, one can obtain other views.

## Competing interests

The authors declare that they have no competing interests.

## Authors' contributions

AH, SA, PW, MO organized the complex data. AK provided secondary structure predictions. NS and PP provided the protein interaction data. PS provided PDB contact data. PW, SA, AH, BG, PP, FB performed analysis. PS, AR, AK, TS, PP, FT, DF supervised the research process and manuscript preparation. All authors read and approved the final manuscript.

## Supplementary Material

Additional File 1 **Description of the protein complex data.** A detailed description of annotated and random complex data used. Click here for file

Additional File 2 **Benchmark of PSIPRED.** A benchmark of PSIPRED's ability to predict secondary structure content from protein sequences is provided. Click here for file

Additional File 3 **Yeast complex complexity and participation distributions.** The distribution of yeast complex complexity and participation approximately follows a power- law. Click here for file

Additional File 4 **Mammalian complex complexity and protein length. **The mean length of proteins in available mammalian complexes is examined with respect to complex complexity. Click here for file

Additional File 5 **Median length of proteins in annotated human complexes.** The median length of proteins in human complexes is examined with respect to complex complexity. Click here for file

Additional File 6 **Yeast complex complexity and protein length.** The mean length of proteins in available yeast complexes is examined with respect to complex complexity. Click here for file

Additional File 7 **Length distribution of human mitochondrial proteins.** The length distribution of human mitochondrial proteins is plotted. Click here for file

Additional File 8 **Random complexes and dN/dS (Human-Mouse Orthologs).** dN/dS ratios of genes associated with model 2 random complexes are examined with respect to complex complexity and participation. Click here for file

Additional File 9 **Binned Plots for Figures 3D and 3F**.3 Click here for file

Additional File 10 **Protein complexes and dN/dS based on orthologs from a variety of species.** dN/dS ratios of genes based on human-dog, human-chimp, human-rat, S. cerevisiae-S. mikatae and S. cerevisiae-S. paradoxus orthologs are examined with respect to complex complexity and participation. Click here for file

Additional File 11 **Complexes and gene conservation.** The fraction of orthologs conserved in yeast and human complexes is examined against complex complexity. Click here for file

Additional File 12 **Extension and subsets of the protein complex data.** Extended yeast complexes, complexes with subcomplexes removed, nuclear and non-nuclear complexes are examined with respect to complex complexity and participation. Click here for file

Additional File 13 **Analysis of protein complex data using the median.** Analysis of protein complex data with respect to mean dN/dS ratios and sequence length is compared with analysis using the median. Click here for file

Additional File 14 **Homogeneity of Protein Properties in Yeast Complexes.** Deviation in pI, secondary structure and evolutionary rate between proteins in annotated yeast complexes are compared to those in random complexes. Click here for file

Additional File 15 **Length difference of interacting proteins.** Large proteins tend to interact with much smaller partners. Click here for file

## References

[B1] Schein CH (1994). Controlling oligomerization of pharmaceutical proteins. Pharm Acta Helv.

[B2] Ali MH, Imperiali B (2005). Protein oligomerization: how and why. Bioorg Med Chem.

[B3] Keeney PM, Xie J, Capaldi RA, Bennett JP (2006). Parkinson's disease brain mitochondrial complex I has oxidatively damaged subunits and is functionally impaired and misassembled. J Neurosci.

[B4] Li B, Samanta A, Song X, Iacono KT, Brennan P, Chatila TA, Roncador G, Banham AH, Riley JL, Wang Q, Shen Y, Saouaf SJ, Greene MI (2007). FOXP3 is a homo-oligomer and a component of a supramolecular regulatory complex disabled in the human XLAAD/IPEX autoimmune disease. Int Immunol.

[B5] Kotzsch A, Nickel J, Seher A, Heinecke K, van Geersdaele L, Herrmann T, Sebald W, Mueller TD (2007). Structure analysis of BMP-2 type I receptor complexes reveals a mechanism of receptor inactivation in juvenile polyposis syndrome. J Biol Chem.

[B6] Fraser HB (2005). Modularity and evolutionary constraint on proteins. Nat Genet.

[B7] Kim PM, Lu LJ, Xia Y, Gerstein MB (2006). Relating three-dimensional structures to protein networks provides evolutionary insights. Science.

[B8] Fornasari MS, Parisi G, Echave J (2007). Quaternary structure constraints on evolutionary sequence divergence. Mol Biol Evol.

[B9] Ruepp A, Brauner B, Dunger-Kaltenbach I, Frishman G, Montrone C, Stransky M, Waegele B, Schmidt T, Noubibou Doudieu O, Stuempflen V, Mewes HW (2008). CORUM: the comprehensive resource of mammalian protein complexes. Nucleic Acids Res.

[B10] Mishra GR, Suresh M, Kumaran K, Kannabiran N, Suresh S, Bala P, Shivakumar K, Anuradha N, Reddy R, Raghavan TM, Menon S, Hanumanthu G, Gupta M, Upendran S, Gupta S, Mahesh M, Jacob B, Mathew P, Chatterjee P, Arun KS, Sharma S, Chandrika KN, Deshpande N, Palvankar K, Raghavnath R, Krishnakanth R, Karathia H, Rekha B, Nayak R, Vishnupriya G, Kumar HG, Nagini M, Kumar GS, Jose R, Deepthi P, Mohan SS, Gandhi TK, Harsha HC, Deshpande KS, Sarker M, Prasad TS, Pandey A (2006). Human protein reference database–2006 update. Nucleic Acids Res.

[B11] Alfarano C, Andrade CE, Anthony K, Bahroos N, Bajec M, Bantoft K, Betel D, Bobechko B, Boutilier K, Burgess E, Buzadzija K, Cavero R, D'Abreo C, Donaldson I, Dorairajoo D, Dumontier MJ, Dumontier MR, Earles V, Farrall R, Feldman H, Garderman E, Gong Y, Gonzaga R, Grytsan V, Gryz E, Gu V, Haldorsen E, Halupa A, Haw R, Hrvojic A, Hurrell L, Isserlin R, Jack F, Juma F, Khan A, Kon T, Konopinsky S, Le V, Lee E, Ling S, Magidin M, Moniakis J, Montojo J, Moore S, Muskat B, Ng I, Paraiso JP, Parker B, Pintilie G, Pirone R, Salama JJ, Sgro S, Shan T, Shu Y, Siew J, Skinner D, Snyder K, Stasiuk R, Strumpf D, Tuekam B, Tao S, Wang Z, White M, Willis R, Wolting C, Wong S, Wrong A, Xin C, Yao R, Yates B, Zhang S, Zheng K, Pawson T, Ouellette BF, Hogue CW (2005). The Biomolecular Interaction Network Database and related tools 2005 update. Nucleic Acids Res.

[B12] Hegyi H, Schad E, Tompa P (2007). Structural disorder promotes assembly of protein complexes. BMC Struct Biol.

[B13] Chen Y, Dokholyan NV (2008). Natural selection against protein aggregation on self-interacting and essential proteins in yeast, fly and worm. Mol Biol Evol.

[B14] Bader GD, Hogue CW (2003). An automated method for finding molecular complexes in large protein interaction networks. BMC Bioinformatics.

[B15] Zhang LV, Wong SL, King OD, Roth FP (2004). Predicting co-complexed protein pairs using genomic and proteomic data integration. BMC Bioinformatics.

[B16] King AD, Przulj N, Jurisica I (2004). Protein complex prediction via cost-based clustering. Bioinformatics.

[B17] Altaf-Ul-Amin M, Shinbo Y, Mihara K, Kurokawa K, Kanaya S (2006). Development and implementation of an algorithm for detection of protein complexes in large interaction networks. BMC Bioinformatics.

[B18] Chua HN, Sung WK, Wong L (2007). Using indirect protein interactions for the prediction of Gene Ontology functions. BMC Bioinformatics.

[B19] Li XL, Foo CS, Ng SK (2007). Discovering protein complexes in dense reliable neighborhoods of protein interaction networks. Comput Syst Bioinformatics Conf.

[B20] Hirsh E, Sharan R (2007). Identification of conserved protein complexes based on a model of protein network evolution. Bioinformatics.

[B21] Lage K, Karlberg EO, Storling ZM, Olason PI, Pedersen AG, Rigina O, Hinsby AM, Tumer Z, Pociot F, Tommerup N, Moreau Y, Brunak S (2007). A human phenome-interactome network of protein complexes implicated in genetic disorders. Nat Biotechnol.

[B22] Fraser HB, Plotkin JB (2007). Using protein complexes to predict phenotypic effects of gene mutation. Genome Biol.

[B23] Zhang B, Park BH, Karpinets T, Samatova NF (2008). From pull-down data to protein interaction networks and complexes with biological relevance. Bioinformatics.

[B24] Qiu J, Noble WS (2008). Predicting co-complexed protein pairs from heterogeneous data. PLoS Comput Biol.

[B25] Friedel CC, Krumsiek J, Zimmer R (2008). Bootstrapping the Interactome: Unsupervised Identification of Protein Complexes in Yeast. RECOMB.

[B26] Sprinzak E, Altuvia Y, Margalit H (2006). Characterization and prediction of protein-protein interactions within and between complexes. Proc Natl Acad Sci USA.

[B27] Mewes HW, Frishman D, Mayer KF, Munsterkotter M, Noubibou O, Pagel P, Rattei T, Oesterheld M, Ruepp A, Stumpflen V (2006). MIPS: analysis and annotation of proteins from whole genomes in 2005. Nucleic Acids Res.

[B28] Güldener U, Münsterkötter M, Kastenmüller G, Strack N, van Helden J, Lemer C, Richelles J, Wodak SJ, García-Martínez J, Pérez-Ortín JE, Michael H, Kaps A, Talla E, Dujon B, André B, Souciet JL, De Montigny J, Bon E, Gaillardin C, Mewes HW (2005). CYGD: the Comprehensive Yeast Genome Database. Nucleic Acids Res.

[B29] Salwinski L, Miller CS, Smith AJ, Pettit FK, Bowie JU, Eisenberg D (2004). The Database of Interacting Proteins: 2004 update. Nucleic Acids Res.

[B30] Chatraryamontri A, Ceol A, Palazzi LM, Nardelli G, Schneider MV, Castagnoli L, Cesareni G (2007). MINT: the Molecular INTeraction database. Nucleic Acids Res.

[B31] Güldener U, Münsterkötter M, Oesterheld M, Pagel P, Ruepp A, Mewes HW, Stümpflen V (2006). MPact: the MIPS protein interaction resource on yeast. Nucleic Acids Res.

[B32] Pagel P, Kovac S, Oesterheld M, Brauner B, Dunger-Kaltenbach I, Frishman G, Montrone C, Mark P, Stuempflen V, Mewes HW, Ruepp A, Frishman D (2005). The MIPS mammalian protein-protein interaction database. Bioinformatics.

[B33] Kerrien S, Alam-Faruque Y, Aranda B, Bancarz I, Bridge A, Derow C, Dimmer E, Feuermann M, Friedrichsen A, Huntley R, Kohler C, Khadake J, Leroy C, Liban A, Lieftink C, Montecchi-Palazzi L, Orchard S, Risse J, Robbe K, Roechert B, Thorneycroft D, Zhang Y, Apweiler R, Hermjakob H (2007). IntAct–open source resource for molecular interaction data. Nucleic Acids Res.

[B34] Finn RD, Marshall M, Bateman A (2005). iPfam: visualization of protein-protein interactions in PDB at domain and amino acid resolutions. Bioinformatics.

[B35] Stein A, Russell RB, Aloy P (2005). 3did: interacting protein domains of known three-dimensional structure. Nucleic Acids Res.

[B36] The Uniprot Consortium (2007). The Universal Protein Resource (UniProt). Nucleic Acids Res.

[B37] Maslov S, Sneppen K (2002). Specificity and stability in topology of protein networks. Science.

[B38] Jones DT (1999). Protein secondary structure prediction based on position-specific scoring matrices. J Mol Biol.

[B39] Pollastri G, Przybylski D, Rost B, Baldi P (2002). Improving the prediction of protein secondary structure in three and eight classes using recurrent neural networks and profiles. Proteins.

[B40] Hubbard TJ, Aken BL, Beal K, Ballester B, Caccamo M, Chen Y, Clarke L, Coates G, Cunningham F, Cutts T, Down T, Dyer SC, Fitzgerald S, Fernandez-Banet J, Graf S, Haider S, Hammond M, Herrero J, Holland R, Howe K, Howe K, Johnson N, Kahari A, Keefe D, Kokocinski F, Kulesha E, Lawson D, Longden I, Melsopp C, Megy K, Meidl P, Ouverdin B, Parker A, Prlic A, Rice S, Rios D, Schuster M, Sealy I, Severin J, Slater G, Smedley D, Spudich G, Trevanion S, Vilella A, Vogel J, White S, Wood M, Cox T, Curwen V, Durbin R, Fernandez-Suarez XM, Flicek P, Kasprzyk A, Proctor G, Searle S, Smith J, Ureta-Vidal A, Birney E (2007). Ensembl 2007. Nucleic Acids Res.

[B41] Wapinski I, Pfeffer A, Friedman N, Regev A (2007). Natural history and evolutionary principles of gene duplication in fungi. Nature.

[B42] Suyama M, Torrents D, Bork P (2006). PAL2NAL: robust conversion of protein sequence alignments into the corresponding codon alignments. Nucleic Acids Res.

[B43] Yang Z (2007). PAML 4: phylogenetic analysis by maximum likelihood. Mol Biol Evol.

[B44] Hirsh AE, Fraser HB, Wall DP (2005). Adjusting for selection on synonymous sites in estimates of evolutionary distance. Mol Biol Evol.

[B45] Resch AM, Carmel L, Mariño-Ramírez L, Ogurtsov AY, Shabalina SA, Rogozin IB, Koonin EV (2007). Widespread positive selection in synonymous sites of mammalian genes. Mol Biol Evol.

[B46] Stajich JE (2007). An Introduction to BioPerl. Methods Mol Biol.

[B47] Liao BY, Zhang J (2008). Null mutations in human and mouse orthologs frequently result in different phenotypes. Proc Natl Acad Sci USA.

[B48] Silva JM, Marran K, Parker JS, Silva J, Golding M, Schlabach MR, Elledge SJ, Hannon GJ, Chang K (2008). Profiling essential genes in human mammary cells by multiplex RNAi screening. Science.

[B49] Schmidt T, Frishman D (2006). PROMPT: a protein mapping and comparison tool. BMC Bioinformatics.

[B50] Barabasi AL, Oltvai ZN (2004). Network biology: understanding the cell's functional organization. Nat Rev Genet.

[B51] Stumpf MP, Wiuf C, May RM (2005). Subnets of scale-free networks are not scale-free: sampling properties of networks. Proc Natl Acad Sci USA.

[B52] Han JD, Dupuy D, Bertin N, Cusick ME, Vidal M (2005). Effect of sampling on topology predictions of protein-protein interaction networks. Nat Biotechnol.

[B53] Wilhelm T, Nasheuer HP, Huang S (2003). Physical and functional modularity of the protein network in yeast. Mol Cell Proteomics.

[B54] Beyer A, Wilhelm T (2005). Dynamic simulation of protein complex formation on a genomic scale. Bioinformatics.

[B55] Wagner GP, Pavlicev M, Cheverud JM (2007). The road to modularity. Nat Rev Genet.

[B56] Pierleoni A, Martelli PL, Fariselli P, Casadio R (2007). eSLDB: eukaryotic subcellular localization database. Nucleic Acids Res.

[B57] Elstner M, Andreoli C, Ahting U, Tetko I, Klopstock T, Meitinger T, Prokisch H (2008). MitoP2: An Integrative Tool for the Analysis of the Mitochondrial Proteome. Mol Biotechnol.

[B58] Gabaldón T, Huynen MA (2007). From endosymbiont to host-controlled organelle: the hijacking of mitochondrial protein synthesis and metabolism. PLoS Comput Biol.

[B59] Bolender N, Sickmann A, Wagner R, Meisinger C, Pfanner N (2008). Multiple pathways for sorting mitochondrial precursor proteins. EMBO Rep.

[B60] Friguet B, Bulteau AL, Petropoulos I (2008). Mitochondrial protein quality control: Implications in ageing. Biotechnol J.

[B61] Young ME, Carroad PAL, Bell RL (1980). Estimation of diffusion coefficients of proteins. Biotechnology and Bioengineering.

[B62] Levy ED, Erba EB, Robinson CV, Teichmann SA (2008). Assembly reflects evolution of protein complexes. Nature.

[B63] Liu G, Uddin M, Islam M, Goodman M, Grossman LI, Romero R, Wildman DE (2007). OCPAT: an online codon-preserved alignment tool for evolutionary genomic analysis of protein coding sequences. Source Code Biol Med.

[B64] Pagel P, Mewes HW, Frishman D (2004). Conservation of protein-protein interactions – lessons from ascomycota. Trends Genet.

[B65] Brown KR, Jurisica I (2007). Unequal evolutionary conservation of human protein interactions in interologous networks. Genome Biol.

[B66] Cai JJ, Woo PC, Lau SK, Smith DK, Yuen KY (2006). Accelerated evolutionary rate may be responsible for the emergence of lineage-specific genes in ascomycota. J Mol Evol.

[B67] Schwartz R, Ting CS, King J (2001). Whole proteome pI values correlate with subcellular localizations of proteins for organisms within the three domains of life. Genome Res.

[B68] Pereira-Leal JB, Levy ED, Kamp C, Teichmann SA (2007). Evolution of protein complexes by duplication of homomeric interactions. Genome Biol.

[B69] Su EC, Chiu HS, Lo A, Hwang JK, Sung TY, Hsu WL (2007). Protein subcellular localization prediction based on compartment-specific features and structure conservation. BMC Bioinformatics.

[B70] Chen Y, Dokholyan NV (2006). The coordinated evolution of yeast proteins is constrained by functional modularity. Trends Genet.

[B71] Schuster-Bockler B, Bateman A (2007). Reuse of structural domain-domain interactions in protein networks. BMC Bioinformatics.

[B72] Urrutia AO, Hurst LD (2003). The signature of selection mediated by expression on human genes. Genome Res.

[B73] Li SW, Feng L, Niu DK (2007). Selection for the miniaturization of highly expressed genes. Biochem Biophys Res Commun.

[B74] Koonin EV, Wolf YI (2006). Evolutionary systems biology: links between gene evolution and function. Curr Opin Biotechnol.

[B75] McInerney JO (2006). The causes of protein evolutionary rate variation. Trends Ecol Evol.

[B76] Pál C, Papp B, Lercher MJ (2006). An integrated view of protein evolution. Nature Rev Genet.

[B77] Rocha EP (2006). The quest for the universals of protein evolution. Trends Genet.

[B78] Drummond DA, Wilke CO (2008). Mistranslation-induced protein misfolding as a dominant constraint on coding-sequence evolution. Cell.

[B79] Zhang J, Maslov S, Shakhnovich EI (2008). Constraints imposed by non-functional protein-protein interactions on gene expression and proteome size. Mol Syst Biol.

[B80] Fraser HB, Hirsh AE, Steinmetz LM, Scharfe C, Feldman MW (2002). Evolutionary rate in the protein interaction network. Science.

[B81] Plotkin JB, Fraser HB (2007). Assessing the determinants of evolutionary rates in the presence of noise. Mol Biol Evol.

[B82] Kim SH, Yi SV (2007). Understanding relationship between sequence and functional evolution in yeast proteins. Genetica.

[B83] Wolf MY, Wolf YI, Koonin EV (2008). Comparable contributions of structural-functional constraints and expression level to the rate of protein sequence evolution. Biol Direct.

[B84] Hamosh A, Scott AF, Amberger JS, Bocchini CA, McKusick VA (2005). Online Mendelian Inheritance in Man (OMIM), a knowledgebase of human genes and genetic disorders. Nucleic Acids Res.

[B85] López-Bigas N, Audit B, Ouzounis C, Parra G, Guigó R (2005). Are splicing mutations the most frequent cause of hereditary disease?. FEBS Lett.

[B86] Wong P, Fritz A, Frishman D (2005). Designability, aggregation propensity and duplication of disease-associated proteins. Protein Eng Des Sel.

[B87] Mika S, Rost B (2006). Protein-protein interactions more conserved within species than across species. PLoS Comput Biol.

