# Supplementary material for: An evolutionary and structural characterization of mammalian protein complex organization
Source: BMC Genomics. 2008 Dec 23;9:629. doi: 10.1186/1471-2164-9-629 (PMC2645396; doi:10.1186/1471-2164-9-629)
Supplement: Additional File 1 — Description of the protein complex data. A detailed description of annotated and random complex data used. [file 1471-2164-9-629-S1.pdf]

Additional file 1: Description of the protein complex data

The complexes collected for this study represent a biased subset of mammalian complexes taken from a variety of species (Figure S1). A large proportion of the complexes localize to the nucleus (Ruepp, et al., 2008). We analyzed the entire set of complexes (ALL) and those found in human (HUMAN). A summary of the complex datasets can be found in Table S1, including the major purification methods used to derive them. In some cases, we also analyzed yeast complexes (YEAST) deposited at MIPS.

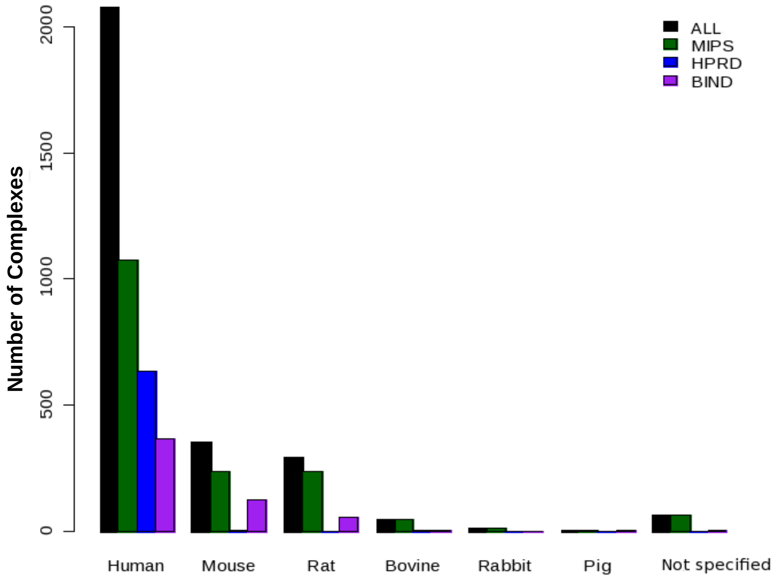

Figure S1: Species distribution of the mammalian complexes
Most complexes in the data are of human origin.

	ALL	HUMAN	YEAST
Number of Complexes	2706	1926	1142
Number of Proteins	4542	2851	2755
Mean (Median) number of Proteins per Complex	4.5 (3)	4.6 (3)	7.8 (4)
Top purification methods	CI – 30.6%<br>AB – 8.8%<br>AF – 8.7%<br>PD – 5.2%<br>CS – 4.5%<br>FW – 2.3%	CI – 23.9%<br>AB – 9.1%<br>AF – 8.8%<br>AT – 7.8%<br>CS – 4.8%<br>PD – 3.9%	CI – 30.1%
% of complexes associated with > 1 purification method	18%	16%	Not available

Table S1. Summary of analyzed complex datasets

The first three rows shows the number of unique complexes, the number of unique proteins and the mean (median) number of proteins per complex in each dataset. The top 7 methods used to purify the complexes is listed for each mammalian complex dataset along with the following code: CI – coimmunoprecipitation, AB - antibaitcoimmunoprecipitation, AT - antitag immunoprecipitation, PD - pulldown, AF - affinitychromatography, CS - cosedimentationthroughdensitygradients, MS - molecularsieving, FW - farwesternblotting
Coimmunoprecipitation is the most common method annotated for the derivation of the complexes.

Completeness of the complex data

By randomly sampling sets of complexes from ones we collected and plotting the number of proteins versus the number of complexes, one can estimate the number of complexes in an organism according to a model. Estimates based on 3 models are reported here: Exponential ($Y = Ae^b$), Power ($Y = Ax^b$) and Quadratic ($Y = Ax^2 + Bx$) where Y = the number of complexes, x = the number of proteins and A, B, b are real constants. We assume that humans have 29000 proteins and yeasts have 6086 proteins. For both human and yeast, a quadratic model fits our data the best. However, because of possible bias in our data, we do not know which estimate is the closest to the actual number of possible complexes in the respective organisms. Nevertheless, the numbers give possible estimates of the completeness of the complexes analyzed which might help guide future research.

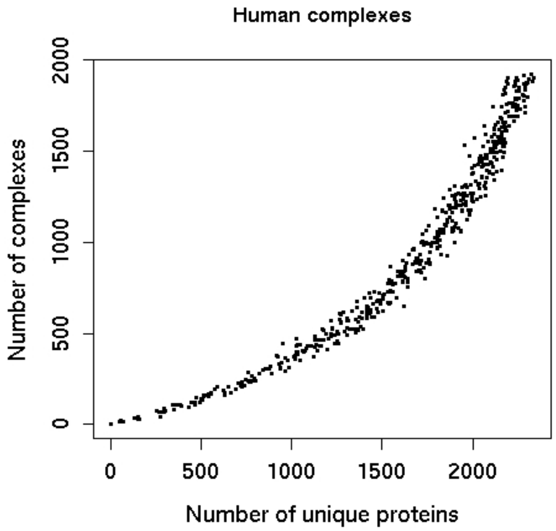

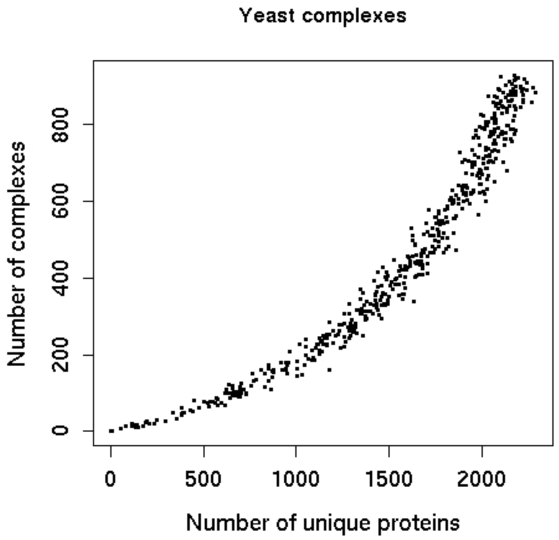

Organism<br>(# of complexes we collected)	Model	Estimated # of different complexes	R^2 for model fit
Human<br>(1926)	$Y = 48.663e^{0.0017x}$	1.25×10^{23}	0.9
	$Y = 0.0442x^{1.3408}$	5100	0.95
	$Y = 0.004x^2 – 0.0566x$	3.3 million	0.98
Yeast<br>(MIPS = 1142,<br>MIPS + Friedel et al. = 2025)	$Y = 30.85e^{0.0017x}$	960 000	0.91
	$Y = 0.038x^{1.2908}$	2900	0.9
	$Y = 0.0002x^2 – 0.0595x$	7200	0.97

The estimated number of complexes for human and yeast are shown in the third column, extrapolated from the plots above. The actual number of human proteins may be much higher than 29000 due to additional presence of isoforms. More accurate models may similarly be generated, taken into account bias in our data.

The Random Complex Data

Random complexes were generated for the purpose of providing a simple screen against spurious trends (ex. Fig. 3C,E) not related to the non-stochastic organization of protein complexes. Random complexes generated in different ways allows one to infer different conclusions when compared to real complexes. If a certain property characterizes both random and real complexes, then significant differences between the two groups of complexes cannot be attributed to the influence of that property alone. If a certain property (ex. randomness) characterizes random complexes but is not found in real complexes, then one can hypothesize that trends significant only to real complexes are associated with either the absence of that property (ex. randomness) and/or the presence of another property specific to real complexes (ex. non-stochastic organization). Below we list two random models tested in the course of this study:

<u>Random model 1: picking proteins with replacement</u>
In the main text of this study we generated random complexes by picking the same number of unique proteins as found in the real complex dataset. Since a protein can participate in multiple complexes, we allowed replacement when proteins were randomly picked. In this way, we simulate greater neutrality in the reuse of proteins in complexes. Characteristics of annotated complexes not found in such random complexes may be attributed to greater restriction to protein reuse in annotated complexes and/or the randomness specific to these random complexes.

<u>Random model 2: picking proteins without replacement</u>
Generating random complexes without adding/subtracting copies of proteins in the network has been commonly accomplished by degree-preserving rewiring, first introduced by Maslov and Sneppen (Science :296(5569):910–913, 2002). Here, the protein-complex network is interpreted as a bipartite graph, i.e. a graph with two distinct sets of nodes (protein nodes and complex nodes), where edges between nodes are only allowed between different kinds. In this context, the two node sets consist of proteins and complexes, and an edge between a protein and a complex indicates membership of a particular protein with a particular complex. In this random model, the number of complexes, the number of unique proteins in each complex and complex participation for each protein (the degree of a protein) is kept the same as in the annotated complexes. The bipartite representation of the complex network is maintained.

The bipartite randomization can be easily achieved: In every randomization step we randomly pick two edges, and exchange their endpoints of one type (either proteins or complexes) without creating multiple edges. This rewiring procedure leads to a loss of degree-correlations between first and second neighbors. Hence, we can observe the degree of randomization by the course of these quantities over the process. This also tells us how many randomization steps we need to perform. In practice, we find that degree-correlations vanish after around one randomization step per edge. So, for our analyses we used five times this number.

Since complex participation is maintained in this random model, we do not observe a difference between Figure 3F and Figure S1cB. Like Figure 3C (Random model 1 complexes), we also are not likely to observe a trend between mean dN/dS values with increasing complex complexity (Figure S1cA), suggesting that the level of protein reuse restriction cannot solely explain the trend between evolutionary rate and complex complexity.

A

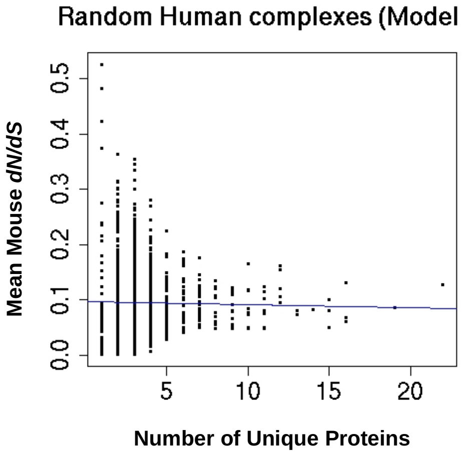

B

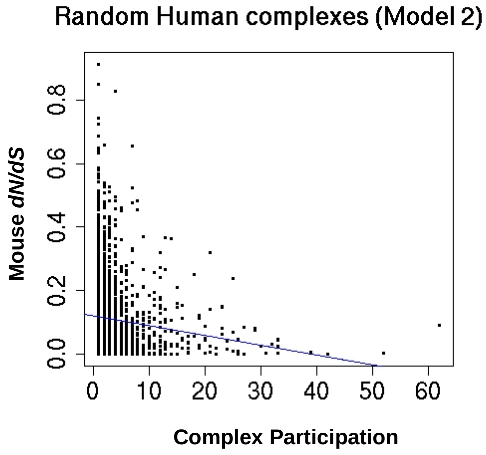

Figure S1c: Model 2 random human complexes where generated. (A) The number of unique proteins in each human complex is plotted against the evolutionary rate of associated genes (dN/dS values of human-mouse orthologs). (B) The same plot as in Figure 3F is observed.

<u>Summary</u>

In both random models, trends between complex complexity and mean dN/dS values were not likely observed. We suggest causes associated with non-stochastic protein complex organization is a major reason for the trend observed in Figure 3D.
