# Supplementary material for: An evolutionary and structural characterization of mammalian protein complex organization
Source: BMC Genomics. 2008 Dec 23;9:629. doi: 10.1186/1471-2164-9-629 (PMC2645396; doi:10.1186/1471-2164-9-629)
Supplement: Additional File 2 — Benchmark of PSIPRED. A benchmark of PSIPRED's ability to predict secondary structure content from protein sequences is provided. [file 1471-2164-9-629-S2.pdf]

Additional file 2: Benchmark of PSIPRED

PSIPRED was benchmarked against the data from Liu & Chou (1999). The Pearson correlation between predicted and benchmark values of secondary structure content (the fraction of residues in coil, sheet and helix in each protein) and the mean absolute error against the benchmark data is shown in the following table:

	Coils	Sheet	Helix
Pearson Correlation	0.8020	0.9234	0.9631
Mean Absolute Error	0.0502	0.0384	0.0396

Table S2. PSIPRED Benchmark.
Helix and sheet content predictions are more accurate than coil predictions.

Reference

Liu W, Chou KC: **Prediction of protein secondary structure content**. *Protein Eng*. 1999, **12**:1041-50.
