# Supplementary material for: An evolutionary and structural characterization of mammalian protein complex organization
Source: BMC Genomics. 2008 Dec 23;9:629. doi: 10.1186/1471-2164-9-629 (PMC2645396; doi:10.1186/1471-2164-9-629)
Supplement: Additional File 3 — Yeast complex complexity and participation distributions. The distribution of yeast complex complexity and participation approximately follows a power- law. [file 1471-2164-9-629-S3.pdf]

Additional file 3: Yeast complex complexity and participation distributions

A

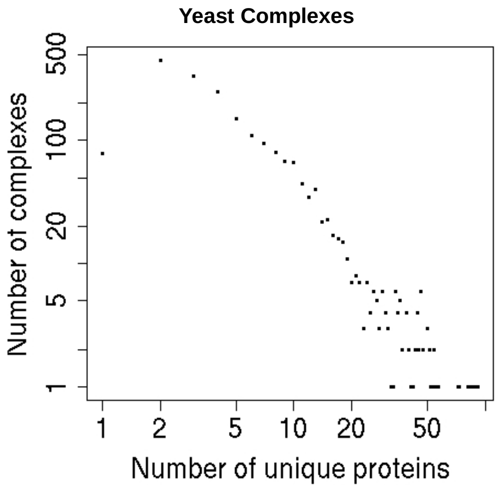

B

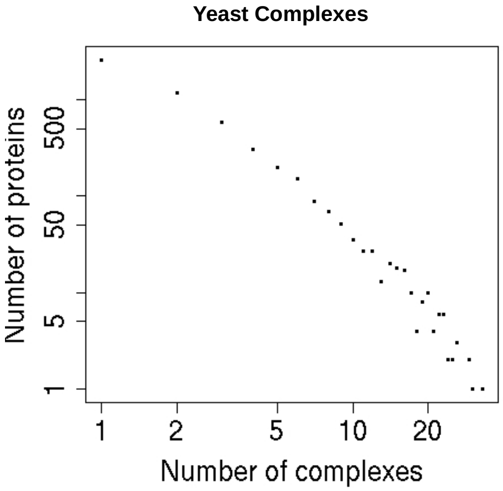

Figure S3. (A) Complex complexity exhibits a broad-tail power law-like distribution when the number of unique proteins increases past a certain point. (B) The number of yeast proteins (y-axis) participating in a particular number of complexes (x-axis) also exhibits a power-law like distribution.
