# Supplementary material for: An evolutionary and structural characterization of mammalian protein complex organization
Source: BMC Genomics. 2008 Dec 23;9:629. doi: 10.1186/1471-2164-9-629 (PMC2645396; doi:10.1186/1471-2164-9-629)
Supplement: Additional File 4 — Mammalian complex complexity and protein length. The mean length of proteins in available mammalian complexes is examined with respect to complex complexity. [file 1471-2164-9-629-S4.pdf]

Additional file 4: Mammalian complex complexity and protein length

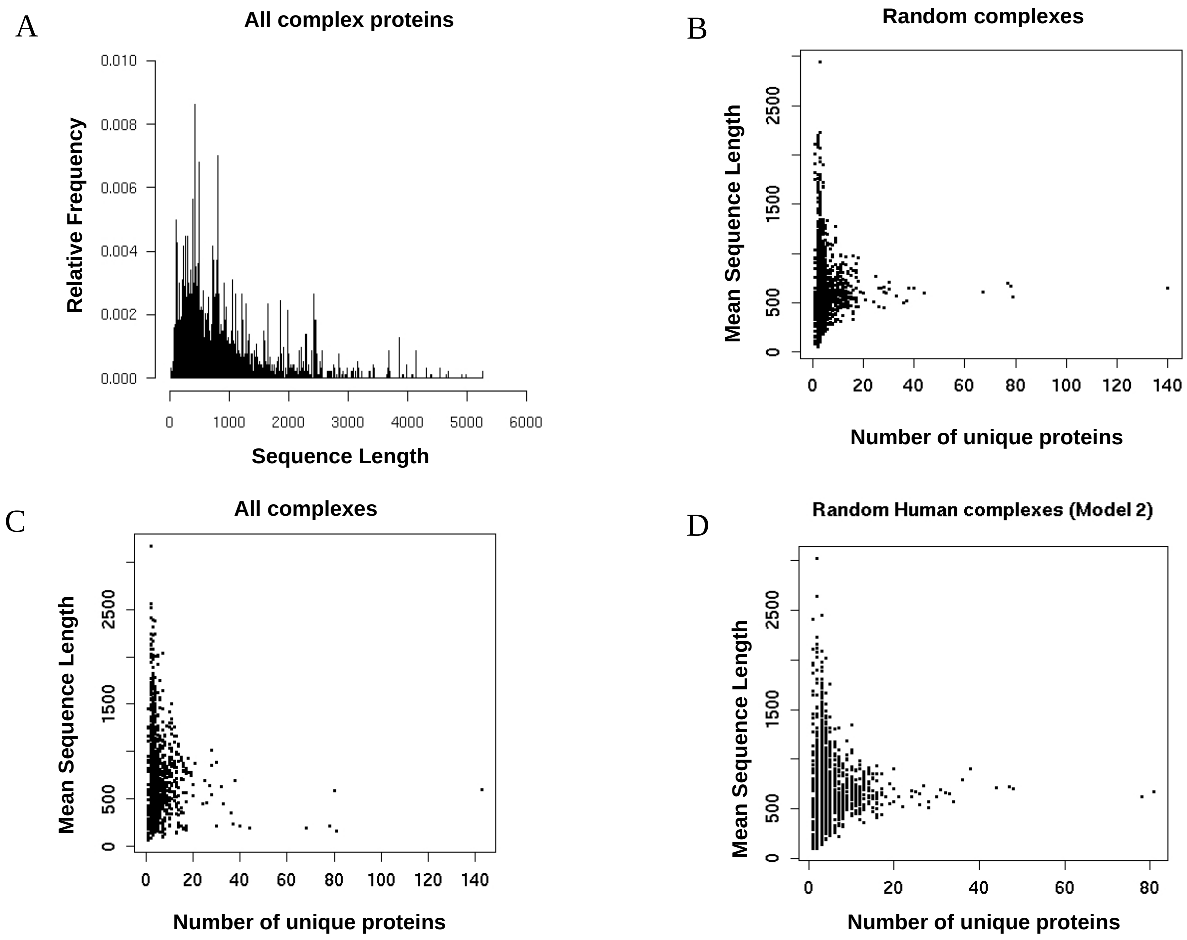

Figure S4. A) Length distribution of proteins in annotated mammalian complexes. Mean lengths of proteins versus the number of unique subunits in: B) Model 1 random mammalian complexes, C) all annotated mammalian complexes. While the mean sequence length stabilizes at a value near ~510aa in model 1 random mammalian complexes, mean sequence length values are much more variable (appearing as scattered points positioned farther away from ~510aa) in real complexes as complex complexity increases. D) Here, we show a similar plot of mean sequence length versus complex complexity for random human complexes (Model 2). In this case, mean sequence length stabilizes at ~620aa as complex complexity increases.
