# Supplementary material for: An evolutionary and structural characterization of mammalian protein complex organization
Source: BMC Genomics. 2008 Dec 23;9:629. doi: 10.1186/1471-2164-9-629 (PMC2645396; doi:10.1186/1471-2164-9-629)
Supplement: Additional File 5 — Median length of proteins in annotated human complexes. The median length of proteins in human complexes is examined with respect to complex complexity. [file 1471-2164-9-629-S5.pdf]

Additional file 5: Median length of proteins in annotated human complexes

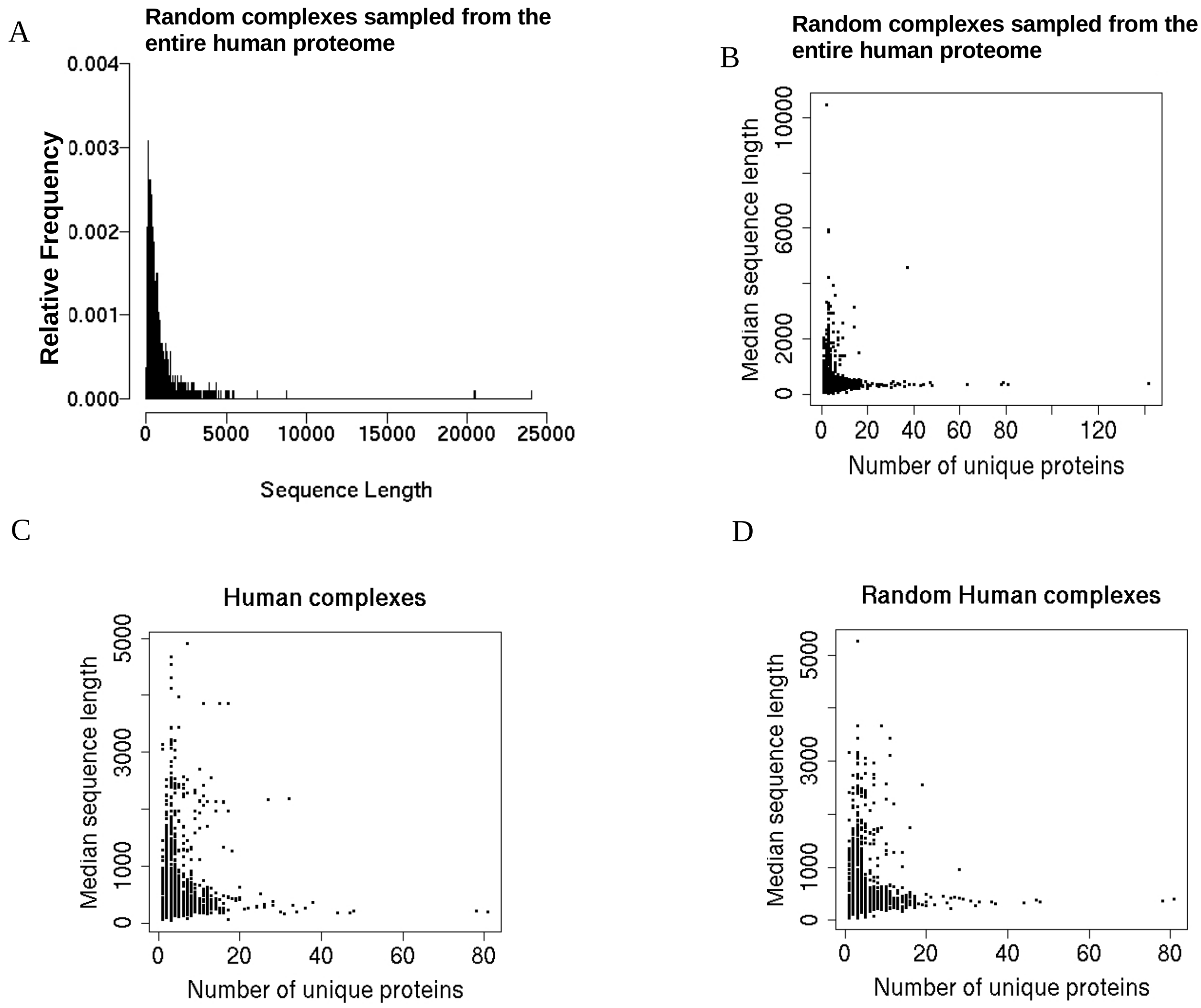

Figure S5. A) Length distribution of proteins in human complexes generated randomly by picking proteins with replacement (Model 1) from the entire human proteome. The median value of the lengths is ~374aa compared to the mean of ~500aa. We plotted also median lengths of proteins versus the number of unique subunits in: B) Model 1 random complexes generated from the entire known human proteome C) annotated human complexes D) Model 1 random human complexes generated from data we collected. Compared with Figure 2, the plots are similar. Figure 2A and Figure S5A are the same. In Figure S5D, when random complexes contain relatively few unique proteins (< 20), the median sequence length is less stable compared with random complexes with more unique proteins (> 20). Like the situation observed in Figure 2C and Figure 2D, there is a larger area where points are scattered in Figure S5C compared with Figure S5D. For complexes with relatively large number of unique subunits (> 40), the median lengths of proteins is quite stable. Hence, Figure S5C and S5D are similar in this region stabilizing at a median sequence length of ~200 aa in Figure S5C and ~380aa in Figure S5D. These results suggest that the median protein length in complexes also reflects to some degree the skewed length distribution of proteins encoded in the proteome (Figure S5A).

Median Length of Proteins in Annotated Human Complexes

E

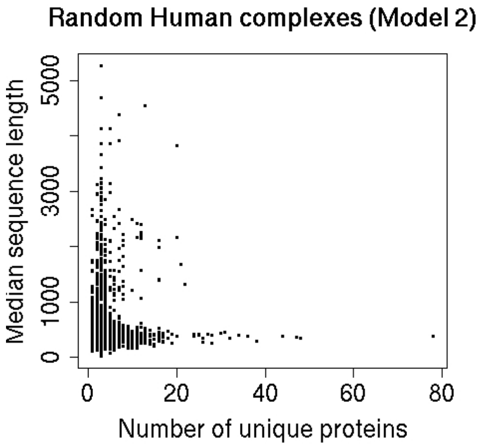

Figure S5-cont. E) This figure is similar to Figure S5D, in which complexes were generated by picking proteins with replacement (Model 1). However, here, we generate Model 2 random complexes. In this random instance, for complexes of high complexity (> 40 unique proteins), median sequence lengths stabilize ~400 aa.
