# Supplementary material for: An evolutionary and structural characterization of mammalian protein complex organization
Source: BMC Genomics. 2008 Dec 23;9:629. doi: 10.1186/1471-2164-9-629 (PMC2645396; doi:10.1186/1471-2164-9-629)
Supplement: Additional File 6 — Yeast complex complexity and protein length. The mean length of proteins in available yeast complexes is examined with respect to complex complexity. [file 1471-2164-9-629-S6.pdf]

Additional file 6: Yeast complex complexity and protein length

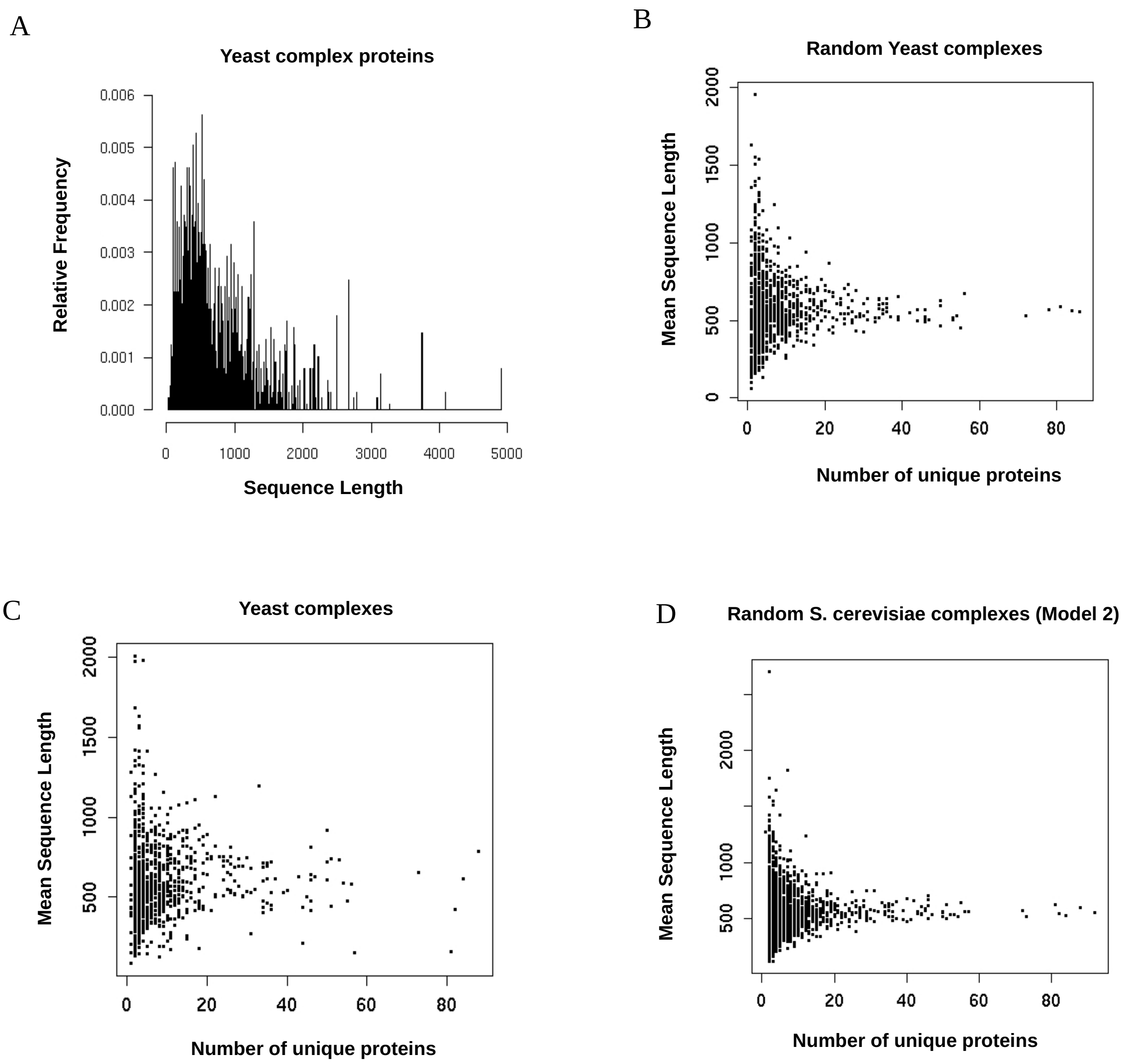

Figure S6. **Mean length of proteins in Yeast Complexes**. A) Length distribution of proteins in the MIPS yeast complexes. Mean lengths of proteins versus the number of unique subunits in: B) random yeast complexes generated with replacement (Model 1) C) MIPS yeast complexes. D) Model 2 random yeast complexes. The mean length of proteins in the yeast complexes are plotted against the complexity of the complexes in terms of the number of unique subunits. While the mean sequence length stabilizes at a value near ~510aa in random yeast complexes (C, D), mean sequence length values are much more variable (appearing as scattered points positioned farther away from ~510aa) in real complexes as complex complexity increases.
