# Supplementary material for: An evolutionary and structural characterization of mammalian protein complex organization
Source: BMC Genomics. 2008 Dec 23;9:629. doi: 10.1186/1471-2164-9-629 (PMC2645396; doi:10.1186/1471-2164-9-629)
Supplement: Additional File 7 — Length distribution of human mitochondrial proteins. The length distribution of human mitochondrial proteins is plotted. [file 1471-2164-9-629-S7.pdf]

Additional file 7: Length distribution of human mitochondrial proteins

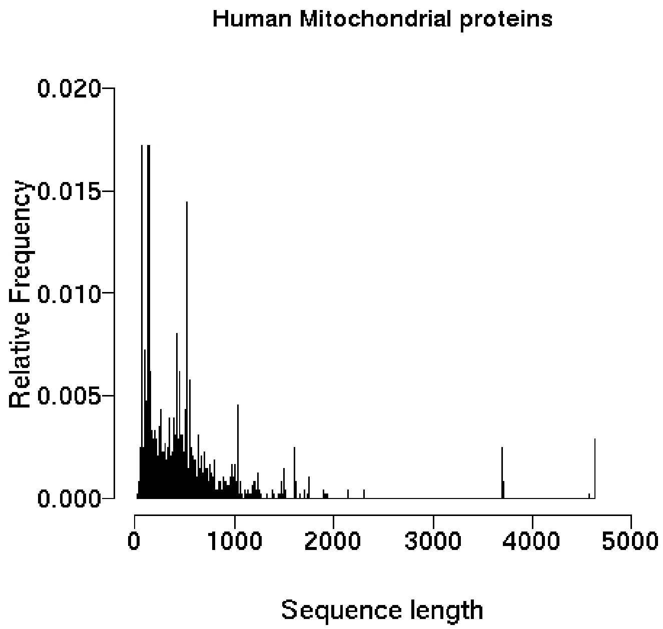

Figure S7. **Human mitochondrial protein length distribution**. The length distribution of mitochondrial proteins from the Eukaryotic Subcellular Localization Database is plotted. Over 97% of the mitochondrial proteins have lengths < 1000 aa.
