# Supplementary material for: An evolutionary and structural characterization of mammalian protein complex organization
Source: BMC Genomics. 2008 Dec 23;9:629. doi: 10.1186/1471-2164-9-629 (PMC2645396; doi:10.1186/1471-2164-9-629)
Supplement: Additional File 8 — Random complexes and dN/dS (Human-Mouse Orthologs). dN/dS ratios of genes associated with model 2 random complexes are examined with respect to complex complexity and participation. [file 1471-2164-9-629-S8.pdf]

Additional file 8: Random Complexes and dN/dS (Human-Mouse Orthologs)

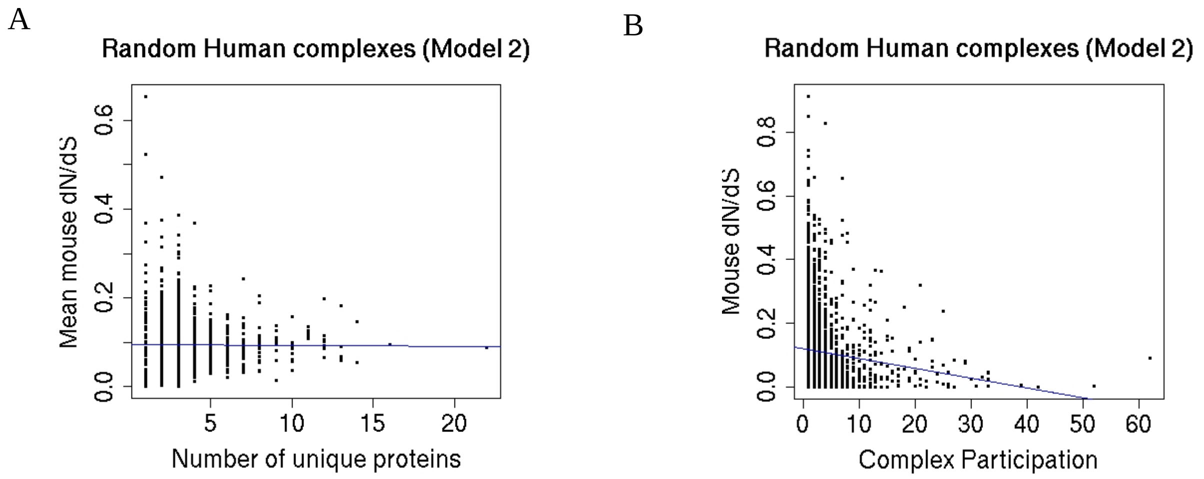

Figure S8 - Random Complexes. A) Model 2 random human complexes were generated. The mean dN/dS ratio between human and mouse orthologs associated with these complexes is plotted against the number of unique proteins in each complex. Trends were rarely observed for such complexes. B) The dN/dS ratios for human-mouse orthologs are plotted against the number of complexes they participate in. Proteins participating in more complexes have significantly lower dN/dS ratios than those with less complex participation (t-test: $P < 7.4 \times 10^{-9}$). Since complex participation of annotated complexes is maintained in Model 2 random complexes, this is the same plot as Figure 3F.
