# Supplementary material for: An evolutionary and structural characterization of mammalian protein complex organization
Source: BMC Genomics. 2008 Dec 23;9:629. doi: 10.1186/1471-2164-9-629 (PMC2645396; doi:10.1186/1471-2164-9-629)
Supplement: Additional File 9 — Binned Plots for Figures 3D and 3F.3 [file 1471-2164-9-629-S9.pdf]

Additional file 9: Binned Plots for Figures 3D and 3F

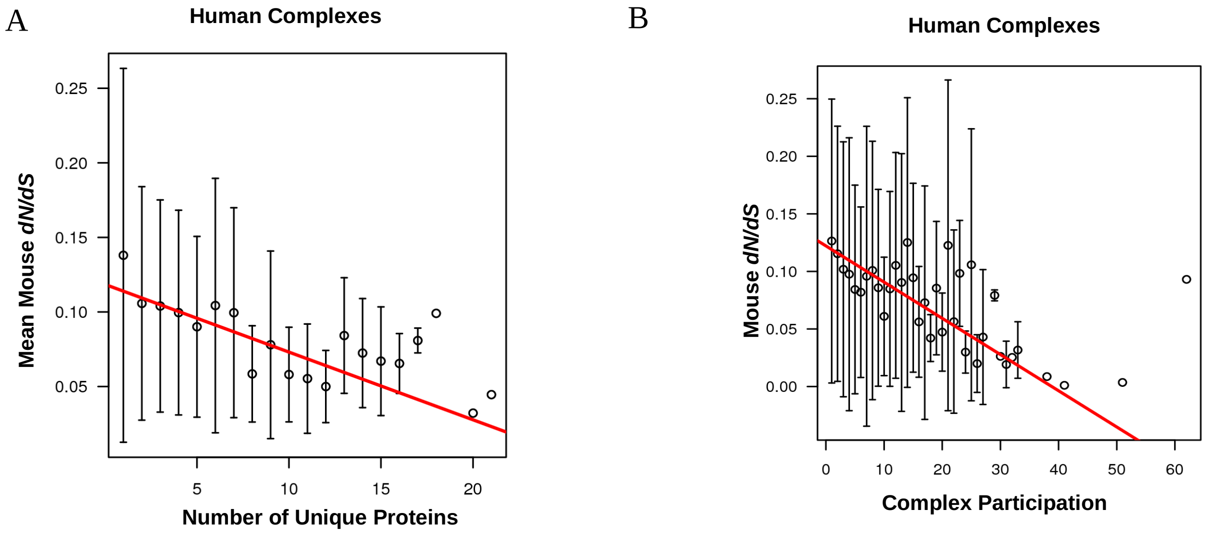

Figure S9 - Binned Plots. Figures S9A and S9B are derived from Figures 3D and 3F of the main text, respectively. The points from the original plots were finely binned (circles representing the mean value at each bin and the bars indicate the first standard deviation). In S9A, mean evolutionary rate tends to decrease with increasing number of unique proteins in the human complexes (t-test: $P < 0.001$). In S9B, the evolutionary rate of proteins tends to decrease with increasing complex participation (t-test: $P < 0.001$).
