# Supplementary material for: An evolutionary and structural characterization of mammalian protein complex organization
Source: BMC Genomics. 2008 Dec 23;9:629. doi: 10.1186/1471-2164-9-629 (PMC2645396; doi:10.1186/1471-2164-9-629)
Supplement: Additional File 10 — Protein complexes and dN/dS based on orthologs from a variety of species. dN/dS ratios of genes based on human-dog, human-chimp, human-rat, S. cerevisiae-S. mikatae and S. cerevisiae-S. paradoxus orthologs are examined with respect to complex complexity and participation. [file 1471-2164-9-629-S10.pdf]

Additional file 10: Protein complexes and dN/dS based on orthologs from a variety of species

Complexes and dN/dS based on Human-Dog Orthologs

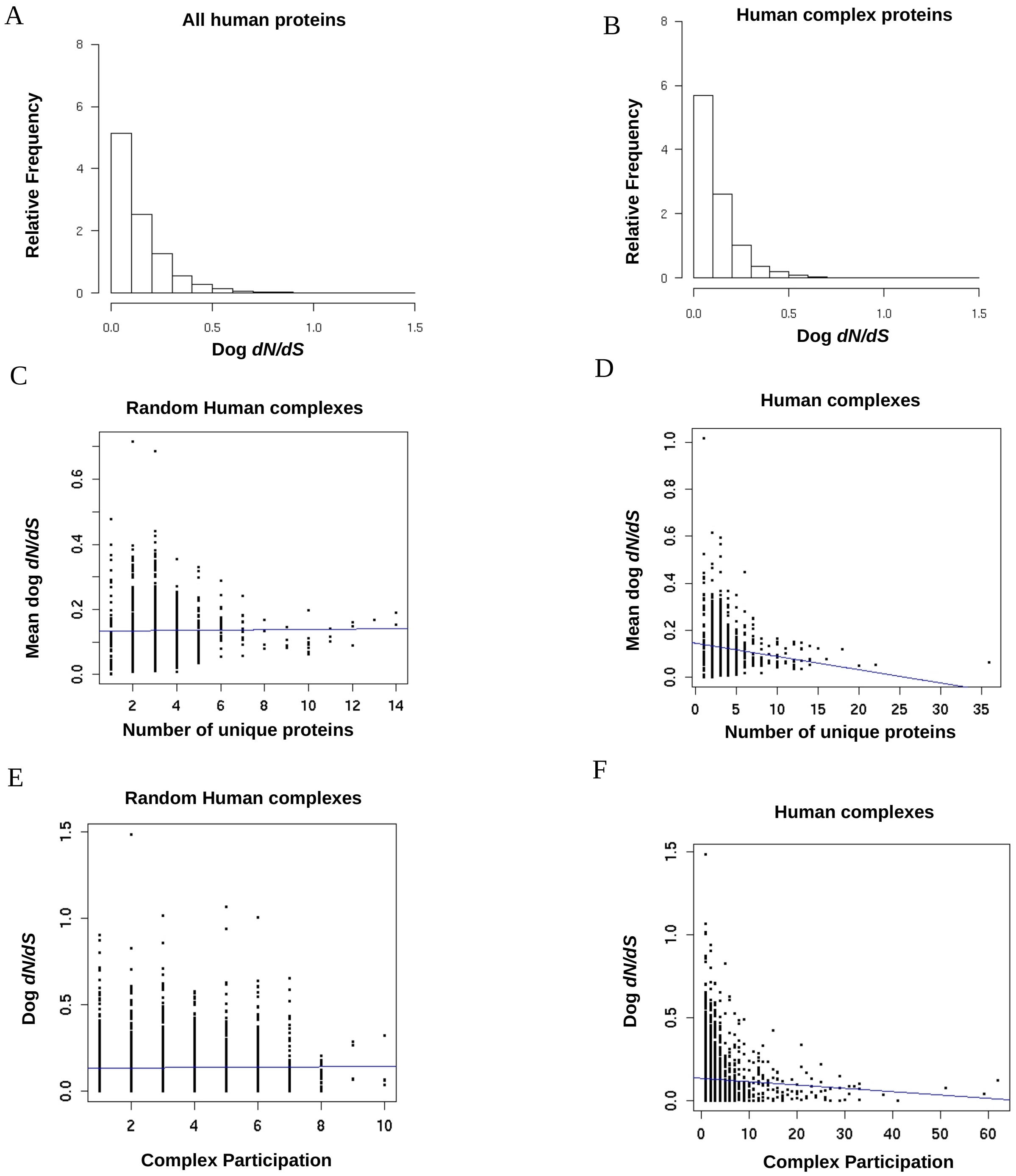

Figure S10a - Complexes and Selection. The dN/dS distribution of genes associated with A) all human proteins and B) proteins in the human complex data. D) The mean dN/dS ratio for human-dog orthologs is plotted against the number of unique proteins for each complex. Complexes with more unique proteins tend to have significantly smaller mean dN/dS ratios than those with less unique proteins (t-test: $P < 4.7 \times 10^{-8}$). F) The dN/dS ratios for human-dog orthologs are plotted against the number of complexes they participate in. Proteins participating in more complexes have significantly lower dN/dS ratios than those with less complex participation (t-test: $P < 2.1 \times 10^{-8}$). C,E) Both trends are rarely observed for Model 1 random complexes.

Complexes and dN/dS based on Human-Chimp Orthologs

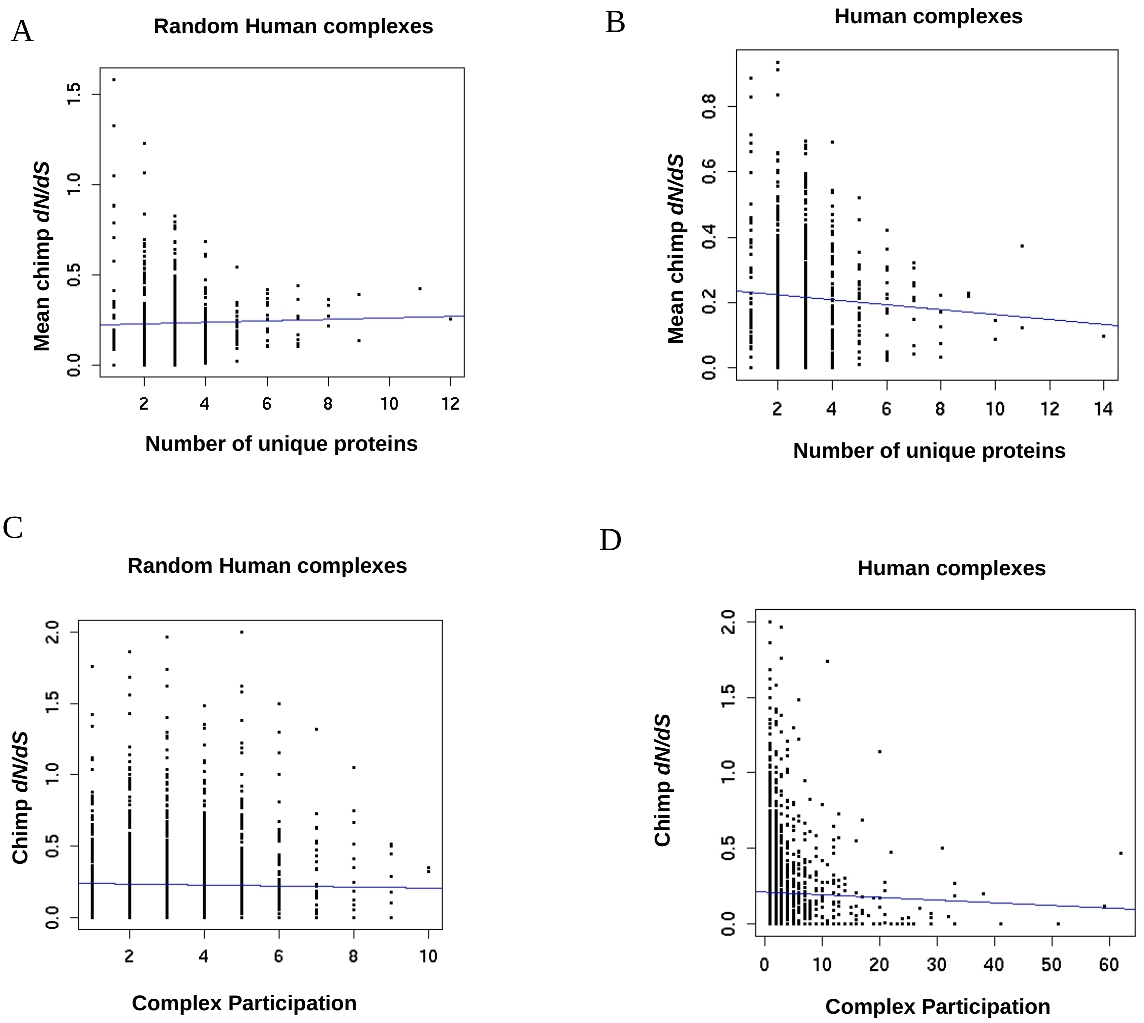

Figure S10b - Complexes and Selection. B) The mean dN/dS ratio for human-chimp orthologs is plotted against the number of unique proteins for each complex. Complexes with more unique proteins were not considered to have significantly smaller mean dN/dS ratios than those with less unique proteins (t-test: $P = 0.1$). D) The dN/dS ratio for human-chimp orthologs is plotted against the number of complexes they participate in. Proteins participating in more complexes have significantly lower dN/dS ratios than those with less complex participation (t-test: $P < 0.0002$). A,C) Such trends were not observed in model 1 random complexes.

Complexes and dN/dS based on Human-Rat Orthologs

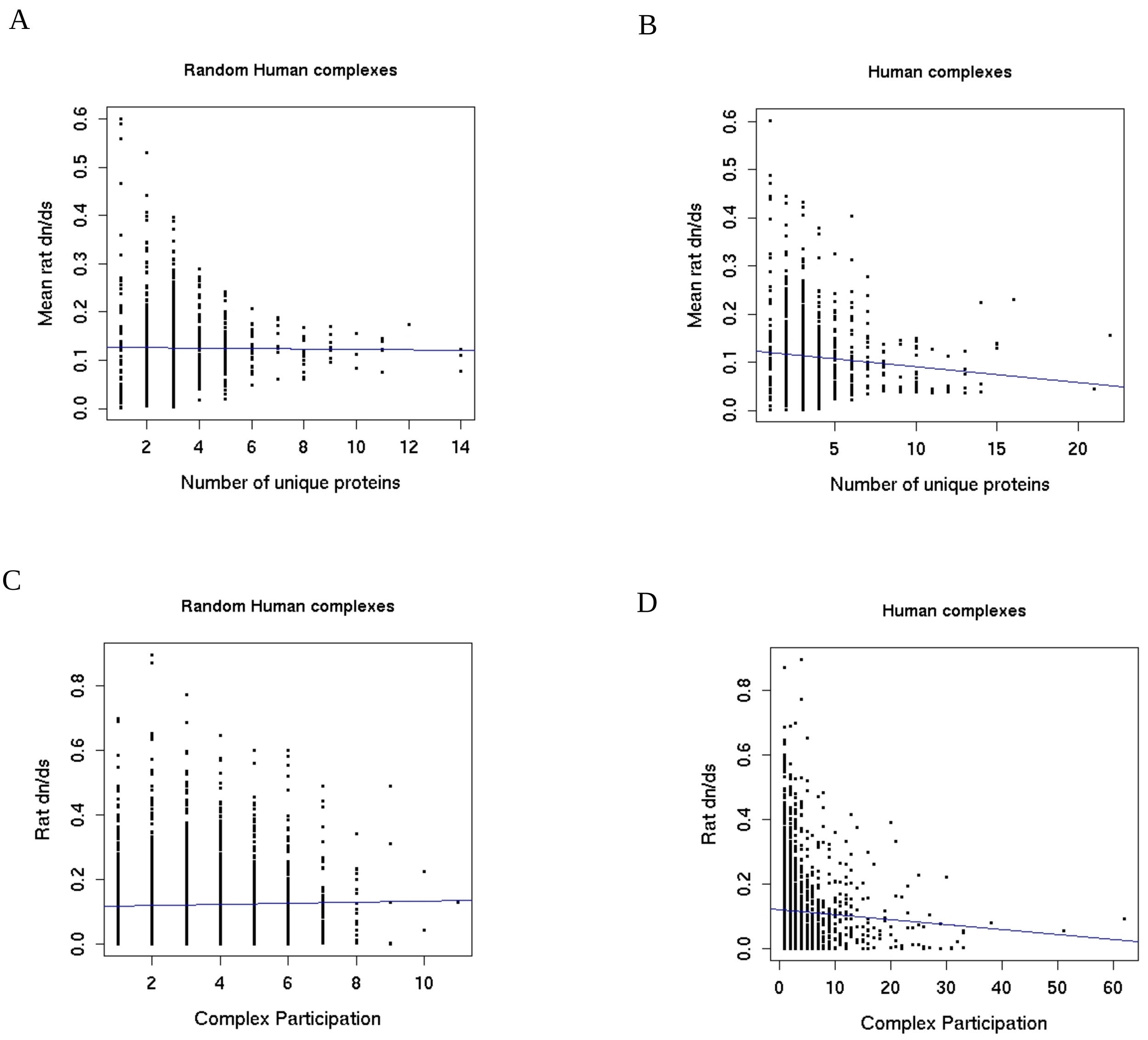

Figure S10c - Complexes and Selection. B) The mean dN/dS ratio for human-rat orthologs is plotted against the number of unique proteins for each complex. Complexes with more unique proteins tend to have significantly smaller mean dN/dS ratios than those with less unique proteins (t-test: $P < 0.002$). D) The dN/dS ratio for human-rat orthologs is plotted against the number of complexes they participate in. Proteins participating in more complexes have significantly lower dN/dS ratios than those with less complex participation (t-test: $P < 2.1 \times 10^{-5}$). A,C) Such trends were not observed in model 1 random complexes.

Complexes and dN/dS (S. cerevisiae-S.mikatae Orthologs)

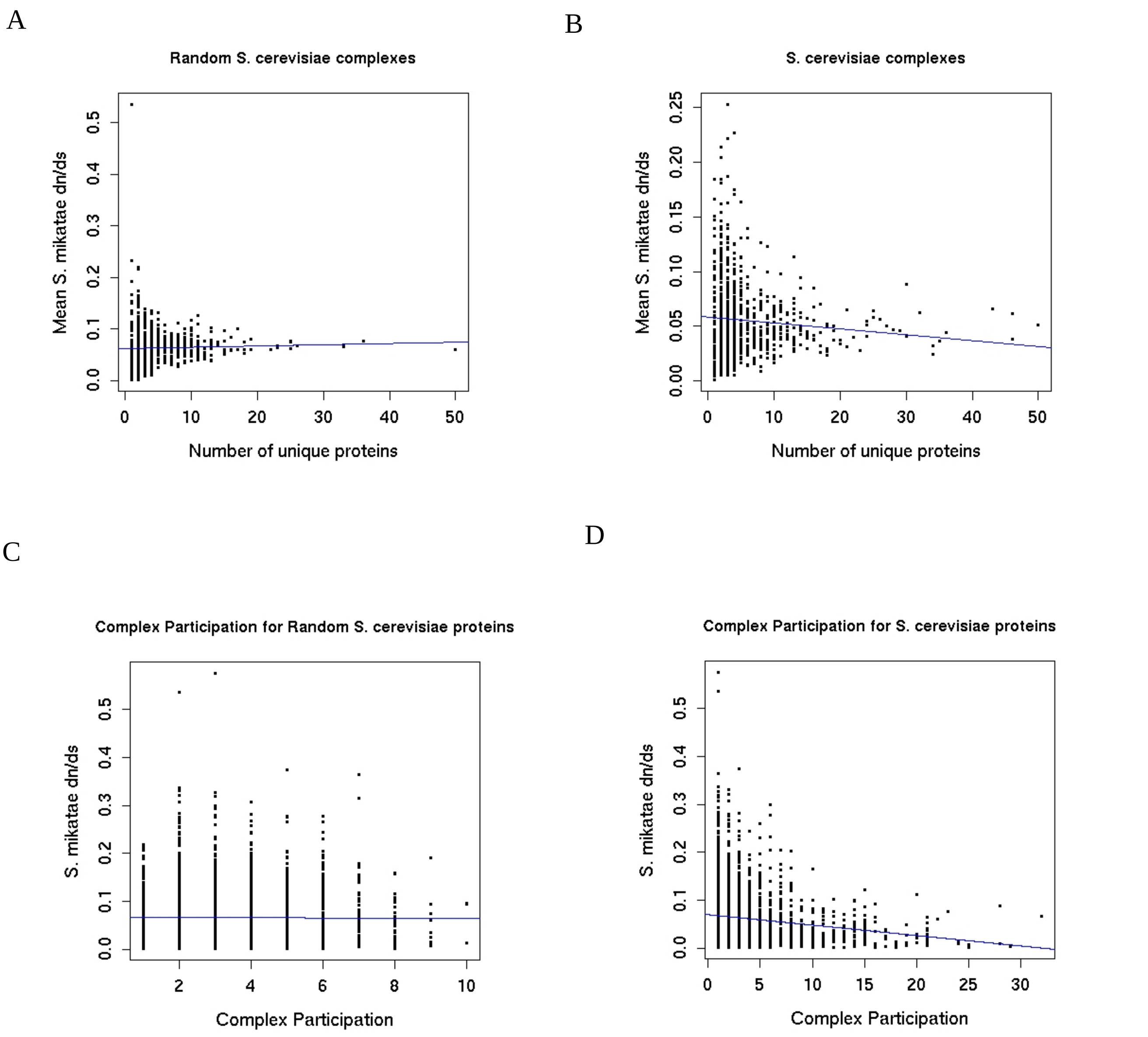

Figure S10d - Complexes and Selection on Yeast Genes. B) The mean dN/dS ratio for S. cerevisiae-S. mikatae orthologs is plotted against the number of unique proteins for each yeast complex. Complexes with more unique proteins tend to have significantly smaller mean dN/dS ratios than those with less unique proteins (t-test: $P < 0.001$). D) The dN/dS ratios for S. cerevisiae-S. mikatae orthologs are plotted against the number of complexes they participate in. Proteins participating in more complexes have significantly lower dN/dS ratios than those with less complex participation (t-test: $P < 2 \times 10^{-16}$). A,C) Such trends were not observed in model 1 random complexes.

Complexes & dN/dS (S.cerevisiae-S.paradoxus Orthologs)

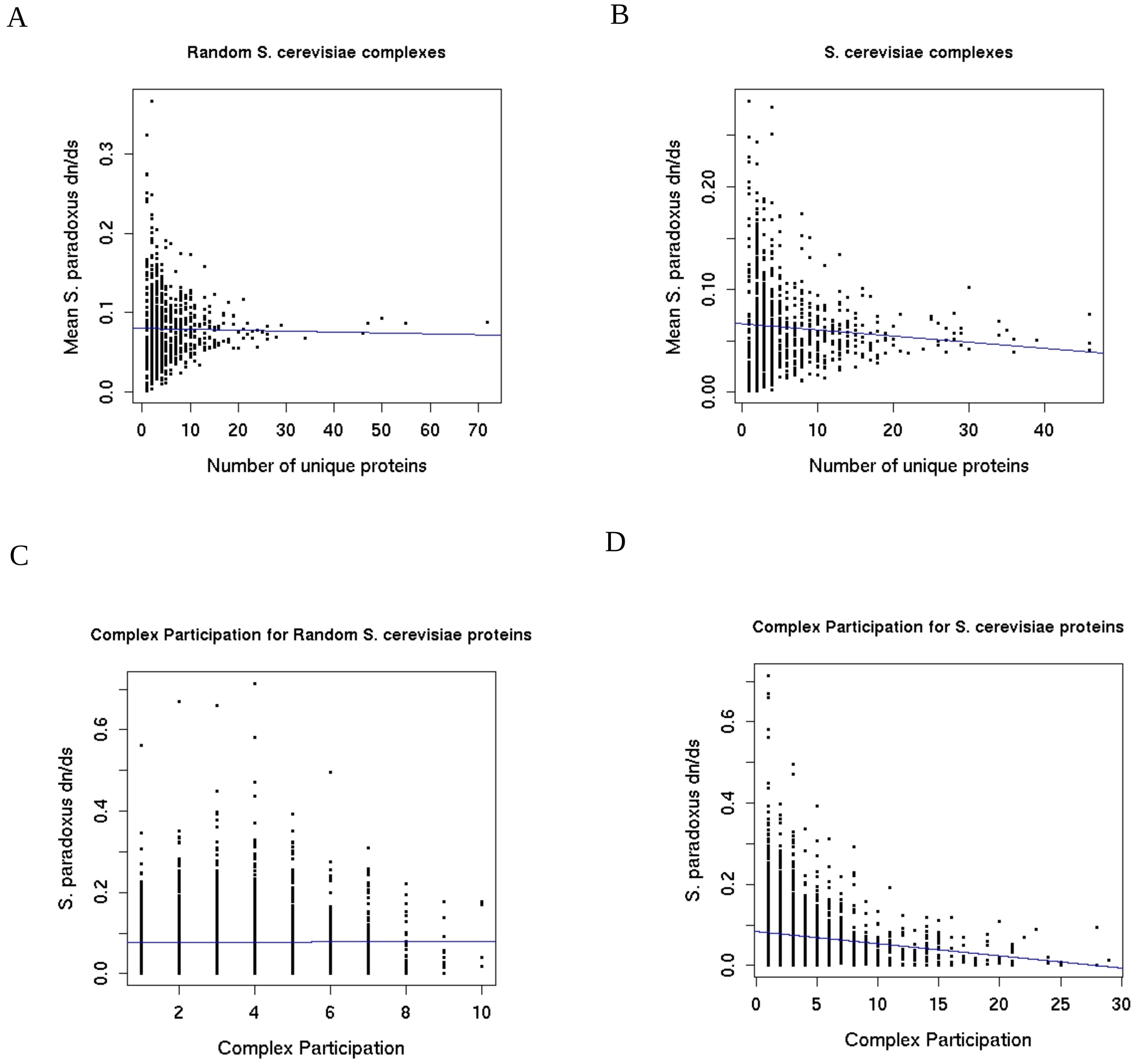

Figure S10e - Complexes and Selection on Yeast Genes. B) The mean dN/dS ratio for S. cerevisiae-S. paradoxus orthologs is plotted against the number of unique proteins for each yeast complex. Complexes with more unique proteins tend to have significantly smaller mean dN/dS ratios than those with less unique proteins (t-test: $P < 0.002$). D) The dN/dS ratios for S. cerevisiae-S. paradoxus orthologs are plotted against the number of complexes they participate in. Proteins participating in more complexes have significantly lower dN/dS ratios than those with less complex participation (t-test: $P < 2 \times 10^{-16}$). A,C) Such trends were not observed in model 1 random complexes.
