# Supplementary material for: An evolutionary and structural characterization of mammalian protein complex organization
Source: BMC Genomics. 2008 Dec 23;9:629. doi: 10.1186/1471-2164-9-629 (PMC2645396; doi:10.1186/1471-2164-9-629)
Supplement: Additional File 11 — Complexes and gene conservation. The fraction of orthologs conserved in yeast and human complexes is examined against complex complexity. [file 1471-2164-9-629-S11.pdf]

# Additional file 11: Complexes and gene conservation

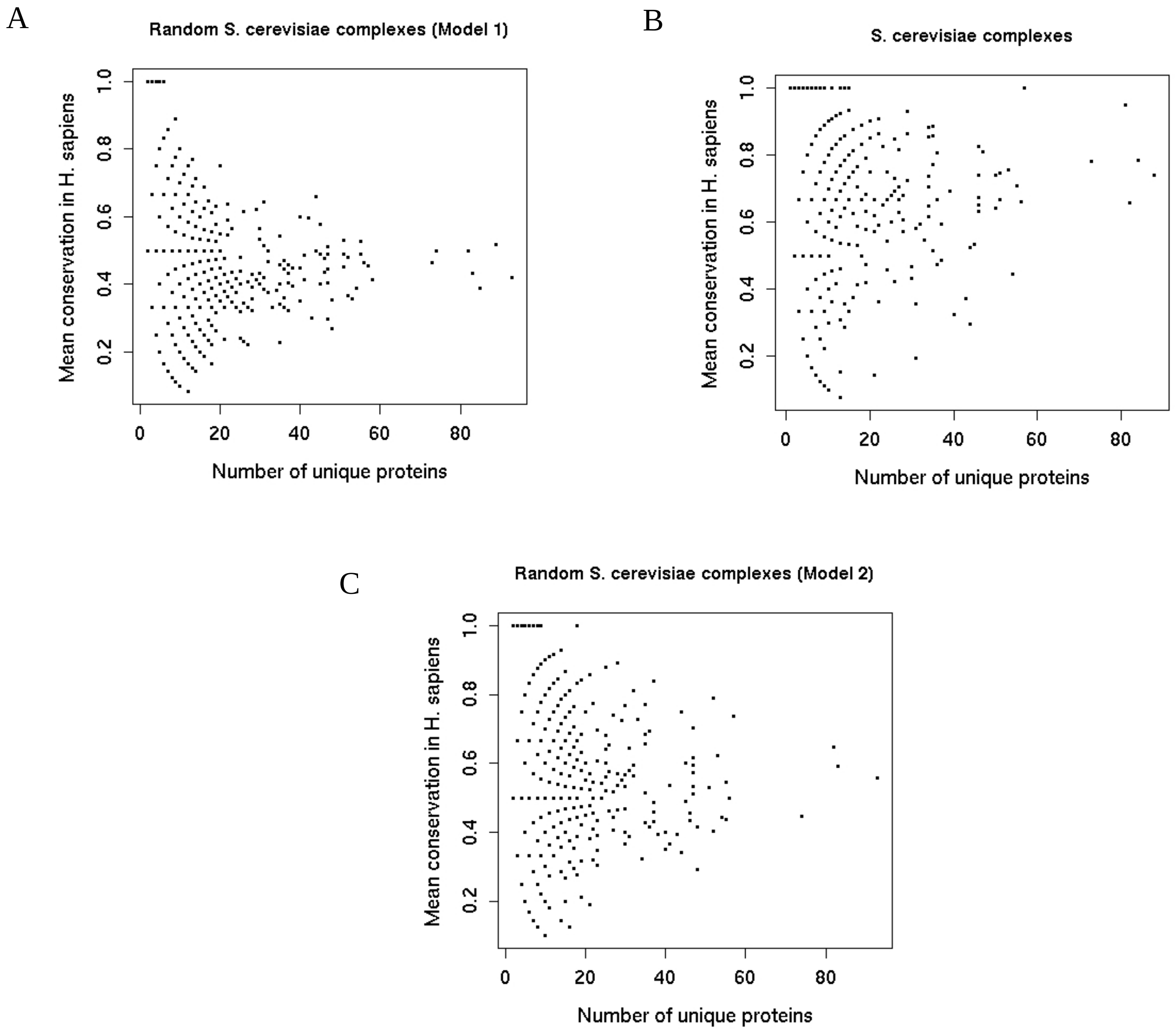


**Figure S11 - Complexes and Conservation.** The fraction of orthologs conserved in yeast and human complexes is plotted against the number of unique subunits in the yeast complex. Unlike for random complexes (A,C), in the annotated complexes (B), highly complex complexes have most of their orthologs conserved.
