# Supplementary material for: An evolutionary and structural characterization of mammalian protein complex organization
Source: BMC Genomics. 2008 Dec 23;9:629. doi: 10.1186/1471-2164-9-629 (PMC2645396; doi:10.1186/1471-2164-9-629)
Supplement: Additional File 12 — Extension and subsets of the protein complex data. Extended yeast complexes, complexes with subcomplexes removed, nuclear and non-nuclear complexes are examined with respect to complex complexity and participation. [file 1471-2164-9-629-S12.pdf]

Additional file 12: Extension and subsets of the protein complex data

Extended Yeast Complex complexity and dN/dS

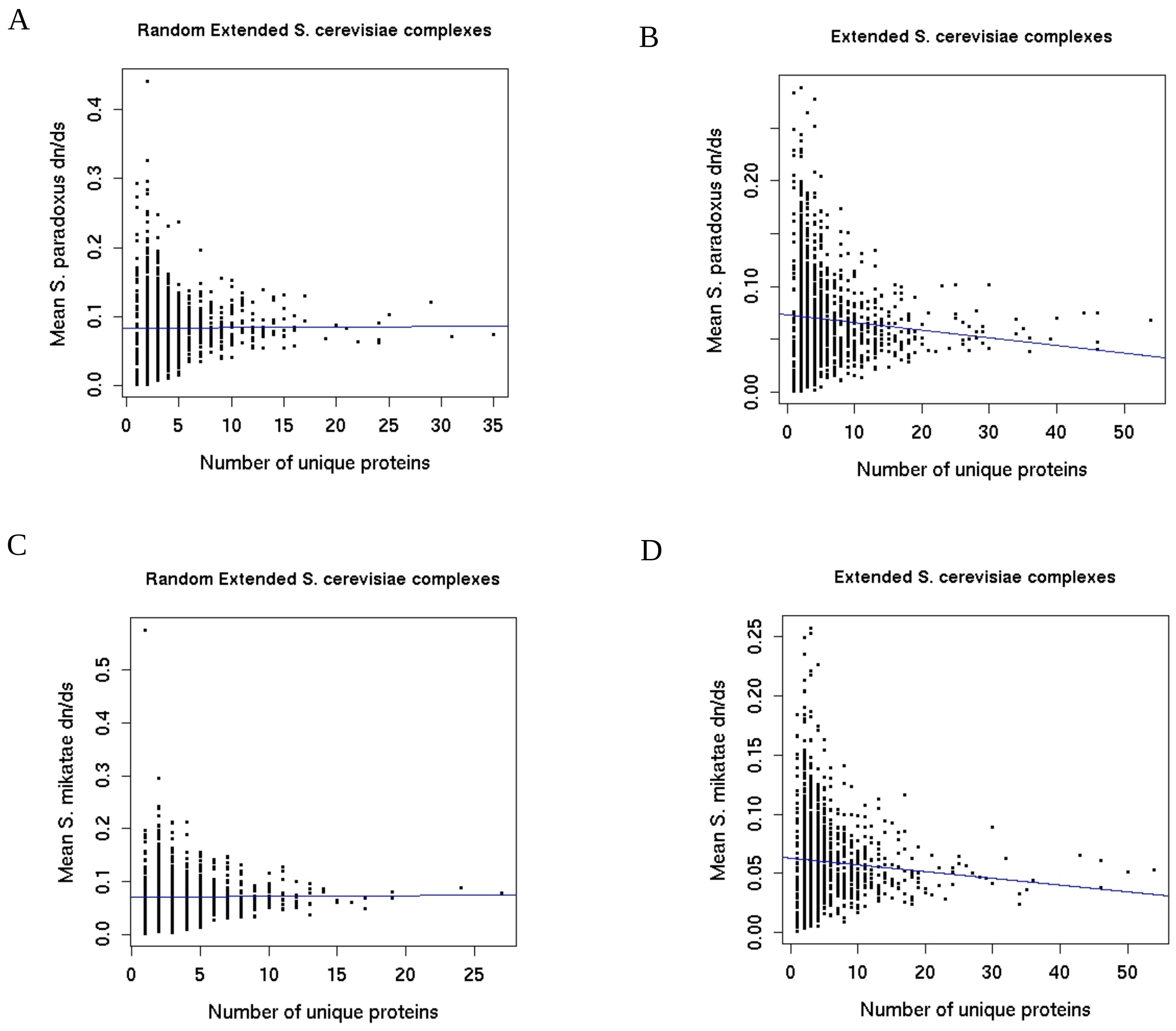

Figure S12 - Complexes and Selection on Yeast Genes. B) The mean dN/dS ratio for S. cerevisiae-S. paradoxus orthologs is plotted against the number of unique proteins for each extended yeast complex. Complexes with more unique proteins tend to have significantly smaller mean dN/dS ratios than those with less unique proteins (t-test: $P < 1.5 \times 10^{-5}$). D) The trend remains present when S. cerevisiae-S. mikatae orthologs were used to compute the dN/dS ratio (t-test: $P < 9.3 \times 10^{-5}$). A,C) Such trends were not observed in model 1 random complexes.

Extended Yeast Complex participation and dN/dS

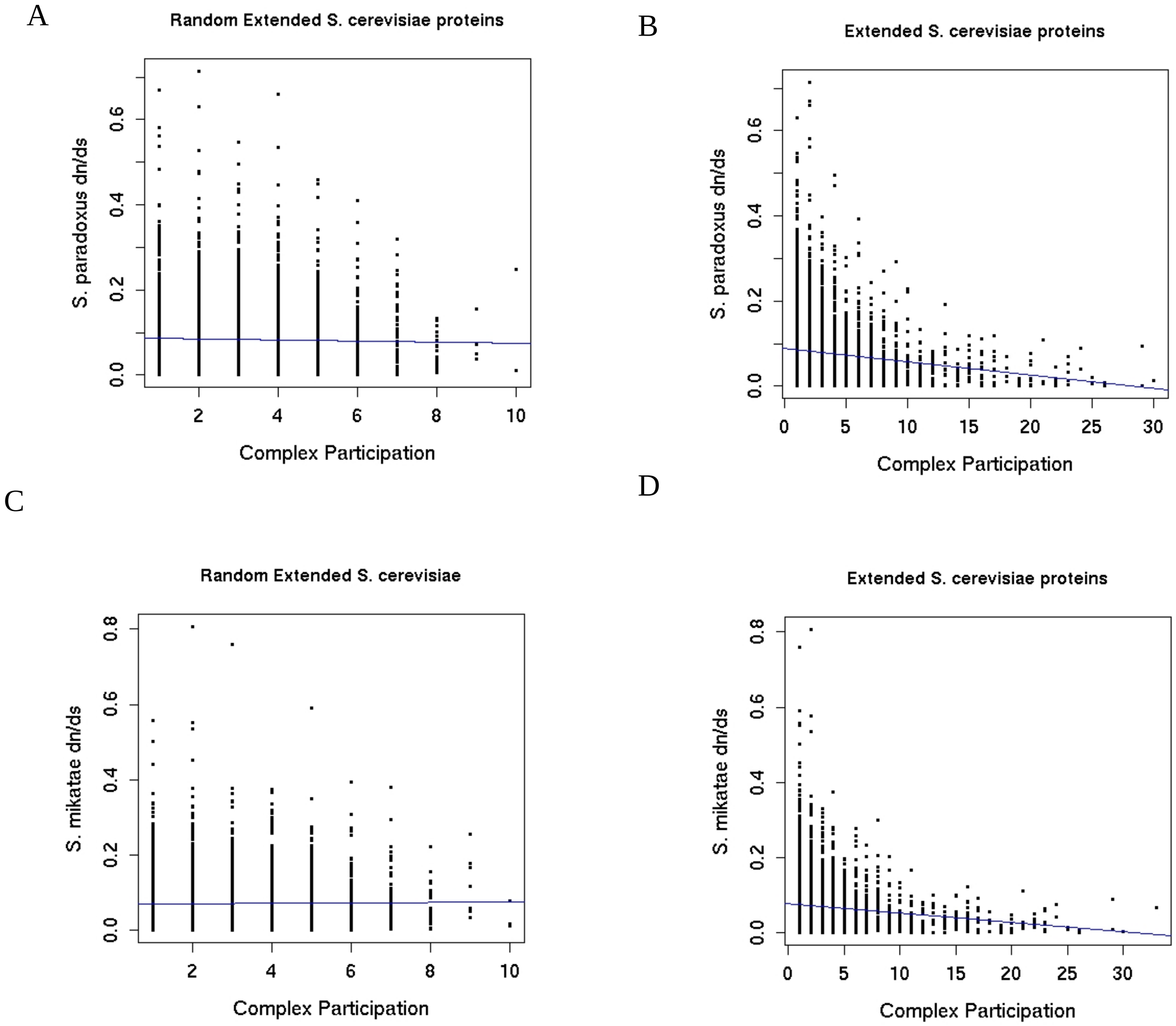

Figure S12b - Complex participation and Selection on Yeast Genes. B) The mean dN/dS ratio from aligning *S. cerevisiae* - *S. paradoxus* orthologs is plotted against the number of extended complexes the *S. cerevisiae* protein was found in. Proteins participating in more complexes tend to have significantly smaller dN/dS ratios than those participating in less complexes (t-test: $P < 2 \times 10^{-16}$). D) We found a similar trend when *S. cerevisiae-S. mikatae* orthologs were used to calculate dN/dS (t-test: $P < 2 \times 10^{-16}$). A,C) Such trends were not observed in model 1 random complexes.

Analysis without subcomplexes

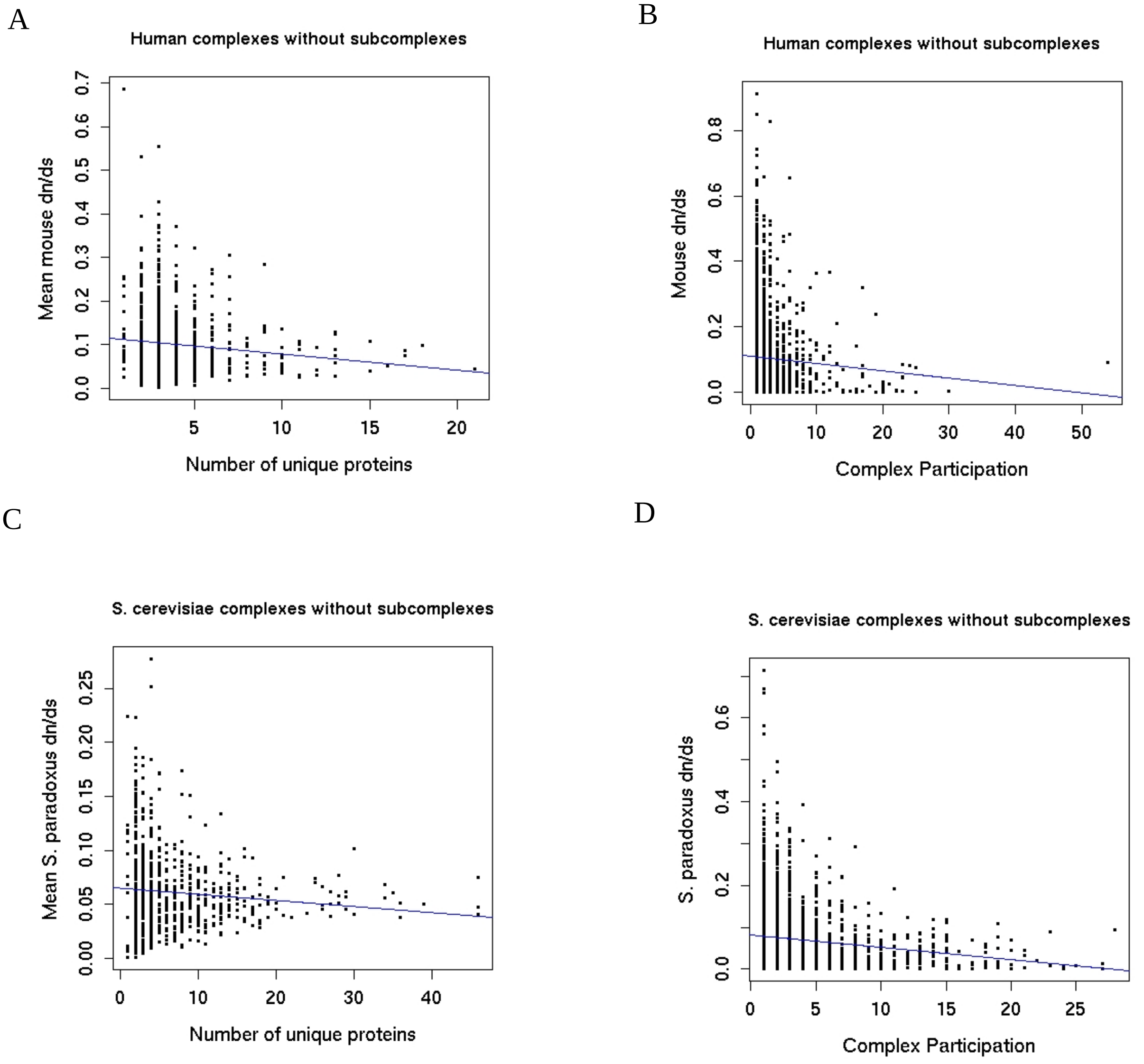

Figure S12c - Analysis without subcomplexes. We re-analyzed the human (A,B) and yeast (C,D) complex data with subcomplexes of complexes removed. A,C) The mean dN/dS ratio tends to decrease with increasing complex complexity (t-test: $P < 0.0002$). B,D) The dN/dS ratio of proteins tends to decrease with increasing complex participation (t-test: $P < 0.0004$).

Nuclear Complexes and dN/dS

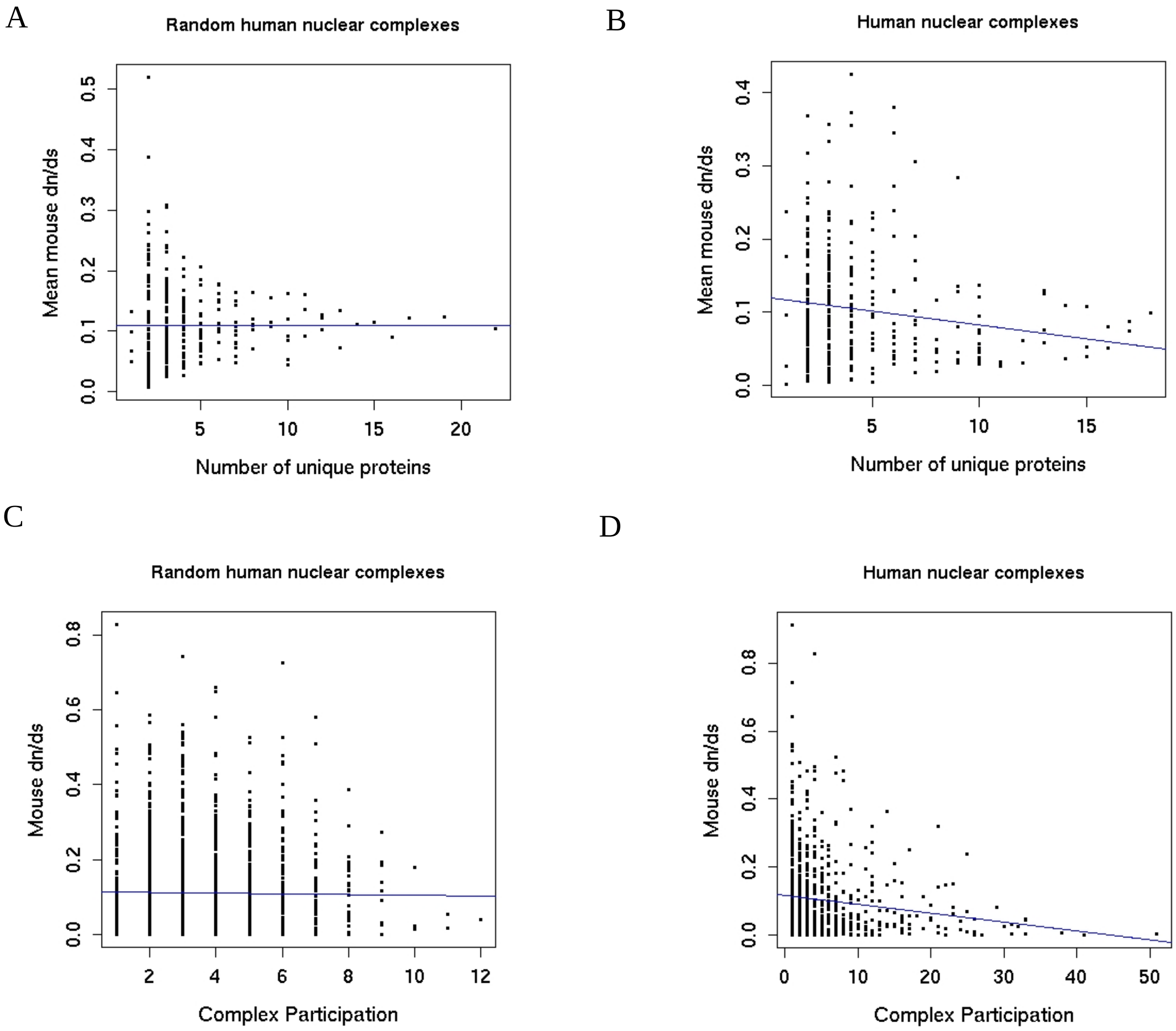

Figure S12d - Nuclear Complexes and Selection on Human Genes. B) The mean dN/dS ratio for human-mouse orthologs is plotted against the number of unique proteins for each human nuclear complex. Complexes with more unique proteins tend to have significantly smaller mean dN/dS ratios than those with less unique proteins (t-test: $P < 0.003$). D) The dN/dS ratio for human-mouse orthologs is plotted against the number of nuclear complexes they participate in. Proteins participating in more complexes have significantly lower dN/dS ratios than those with less complex participation (t-test: $P < 1.9 \times 10^{-6}$). A,C) Such trends were seldomly observed for model 1 randomly-generated complexes.

Non-Nuclear Complexes and dN/dS

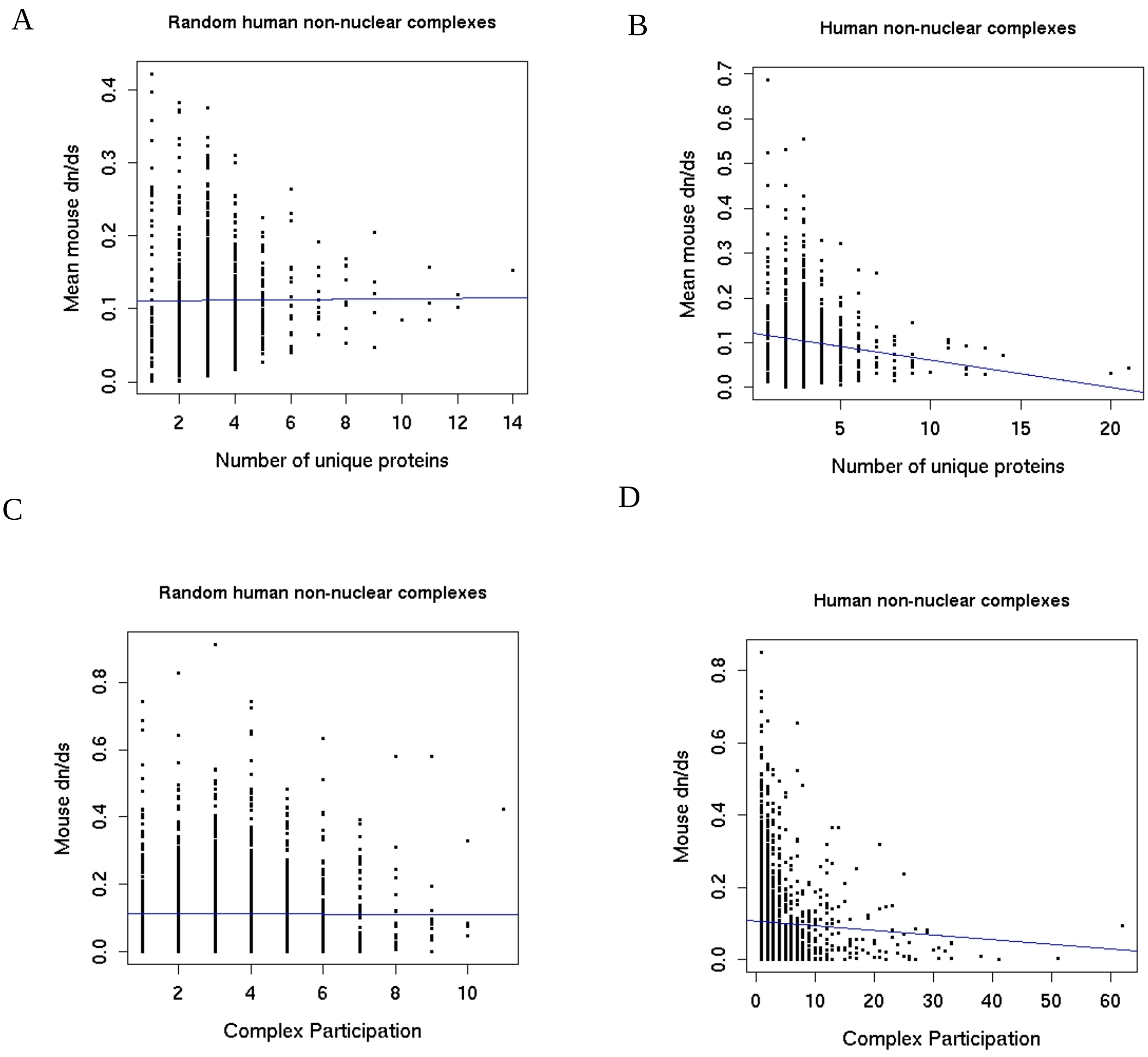

Figure S12e - Non-Nuclear Complexes and Selection on Human Genes. B) The mean dN/dS ratio for human-mouse orthologs is plotted against the number of unique proteins for each human non-nuclear complex. Complexes with more unique proteins tend to have significantly smaller mean dN/dS ratios than those with less unique proteins (t-test: $P < 4.1 \times 10^{-6}$). D) The dN/dS ratio for human-mouse orthologs is plotted against the number of non-nuclear complexes they participate in. Proteins participating in more complexes have significantly lower dN/dS ratios than those with less complex participation (t-test: $P < 1.3 \times 10^{-8}$). A,C) Such trends were seldomly observed for model 1 randomly-generated complexes.
