# Supplementary material for: An evolutionary and structural characterization of mammalian protein complex organization
Source: BMC Genomics. 2008 Dec 23;9:629. doi: 10.1186/1471-2164-9-629 (PMC2645396; doi:10.1186/1471-2164-9-629)
Supplement: Additional File 13 — Analysis of protein complex data using the median. Analysis of protein complex data with respect to mean dN/dS ratios and sequence length is compared with analysis using the median. [file 1471-2164-9-629-S13.pdf]

Additional file 13: Analysis of protein complex data using the median

As measures of central tendency, information is lost when one uses either the mean or median. The mean transforms many data points into a single summarizing datum. The median, ignores all data except the middle data points (taking the mean of the two middle points when there is an even number of data). The mean reflects the data at hand much more than the median but may change significantly when outlying data points are added or subtracted from the data. The median is much more robust to changes to the data. However, conclusions must recognize information loss, especially when medians are in use.

In this study, we observed that the mean *dN/dS* ratios of proteins in the collected complexes tend to decrease with increasing complex complexity. There seems to be at least two major phenomena associated with this observation:

1) The skewed distribution of *dN/dS* ratios amongst genes as depicted in Figure 3B. In particular, as complex complexity increases, the mean *dN/dS* ratios of proteins in complexes tend to stabilize towards the mean of this skewed distribution.

2) increased selective pressure on proteins involved in the organization and function of more complex complexes

Interestingly, we also observed such trends for randomly generated yeast and mammalian complexes using median *dN/dS* ratios but not the mean (S13a, c). We, likewise, observed trends when we plotted median sequence length versus complex complexity (S13b, d) for random complexes but such trends were not observed when mean sequence length was plotted. Since random complexes are by definition much less organized than annotated complexes, trends observed with median values in the random complex plots are more likely due to sampling from a skewed distribution of values rather than selective pressure associated with the organization of proteins into complexes. Unlike mean values which summarizes all *dN/dS* values in a complex, medians capture only the middle values in each complex. Thus, trends derived from medians can be more sensitive to the skewness of the sampled values compared with information associated with all proteins contained in each complex. In fact, when median values are analyzed, trends for random complexes could have lower P-values than those from annotated complexes (S13b, d). Thus, as statistical estimators, our results suggest that means and medians can reflect differences between the two factors ((1)skewness of the sampled data and (2) grouping of proteins into complexes) which have contributed to the observed trends in the random complexes.

For the annotated yeast and mammalian complex data available, we found a consistent negative correlation between mean or median *dN/dS* ratios with complex complexity (Figures S13a-A, B, S13c-A, B). Observed trends between mean or median sequence length and complex complexity were not found to be consistent (Figures S13b-A, B, S13d-A, B), although there seems to be a boundary in the upper right corner of the plots where data points are never found.

S13a – Analysis of Human Complex Complexity and dN/dS using the median

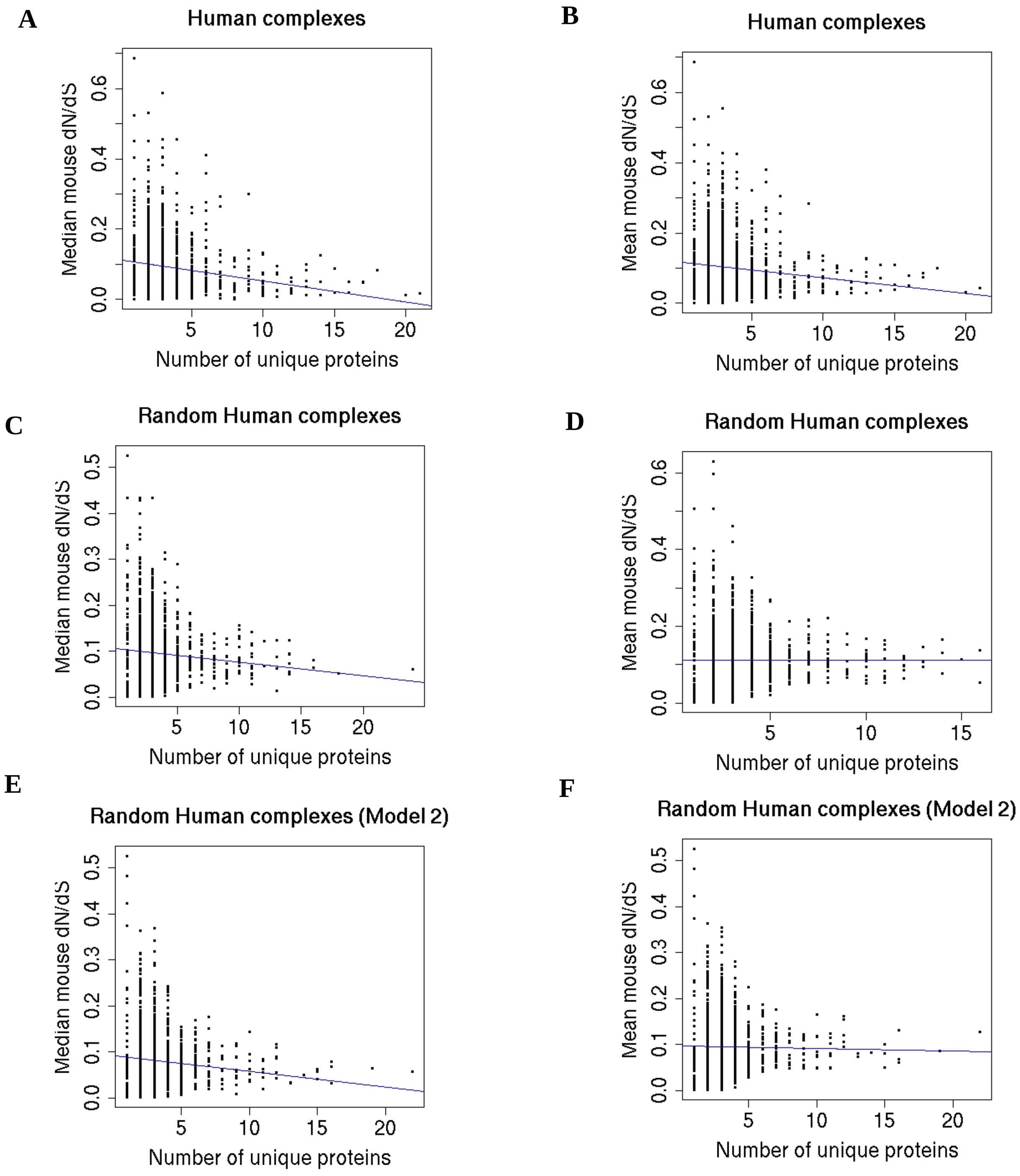

Figure S13a: Data from Figures 3C and 3D are analyzed. Median dN/dS values from human-mouse orthologs tend to decrease with increasing complex complexity for A) annotated complexes (t-test: $P < 1.4 \times 10^{-11}$), C) Model 1 random complexes (t-test: $P < 7 \times 10^{-4}$), and E) Model 2 random complexes (t-test: $P < 4.8 \times 10^{-5}$). In contrast, significant trends were only observed for B) annotated complexes when mean dN/dS ratios were plotted (Figure 3D -> t-test: $P < 3.1 \times 10^{-7}$).

S13b – Analysis of Human Complex Complexity and protein length using the median

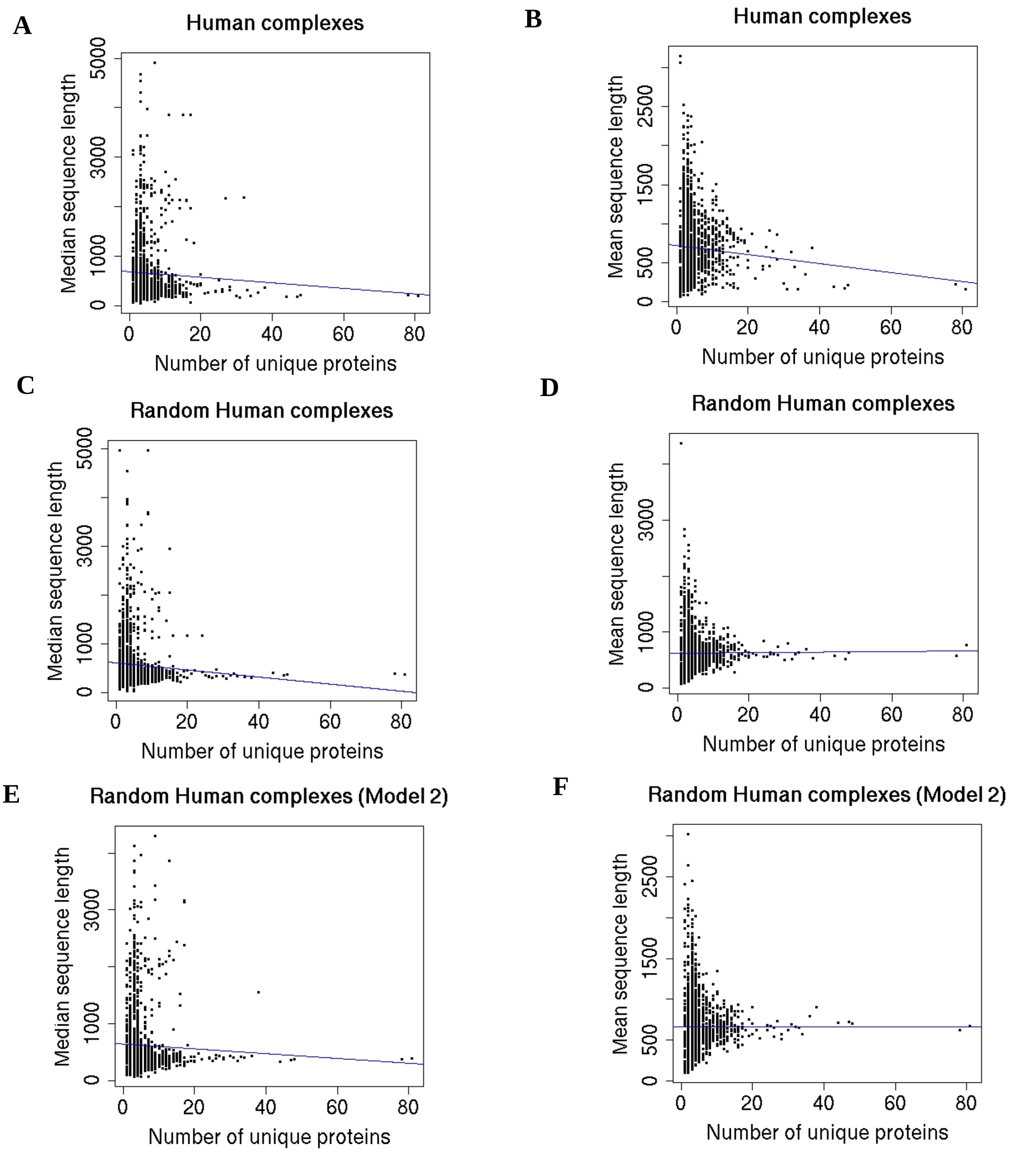

Figure S13b: Data from Figures 2C and 2D are re-analyzed. Median sequence length tend to decrease with increasing complex complexity for A) annotated complexes (t-test: $P < 0.06$) C) Model 1 random complexes (t-test: $P < 0.004$) and E) Model 2 random complexes (t-test: $P < 0.13$). The situation here is of note because random complexes were observed to have an even more significant trend than annotated complexes. Although the trend for plot (E) is considered insignificant, P-values for such plots have been observed to be < 0.03. When mean sequence length is plotted against complex complexity, we observe a significant trend for B) annotated complexes (t-test: $P < 0.002$), but not D,F) the random complexes.

S13c – Analysis of Yeast Complex Complexity and dN/dS using the median

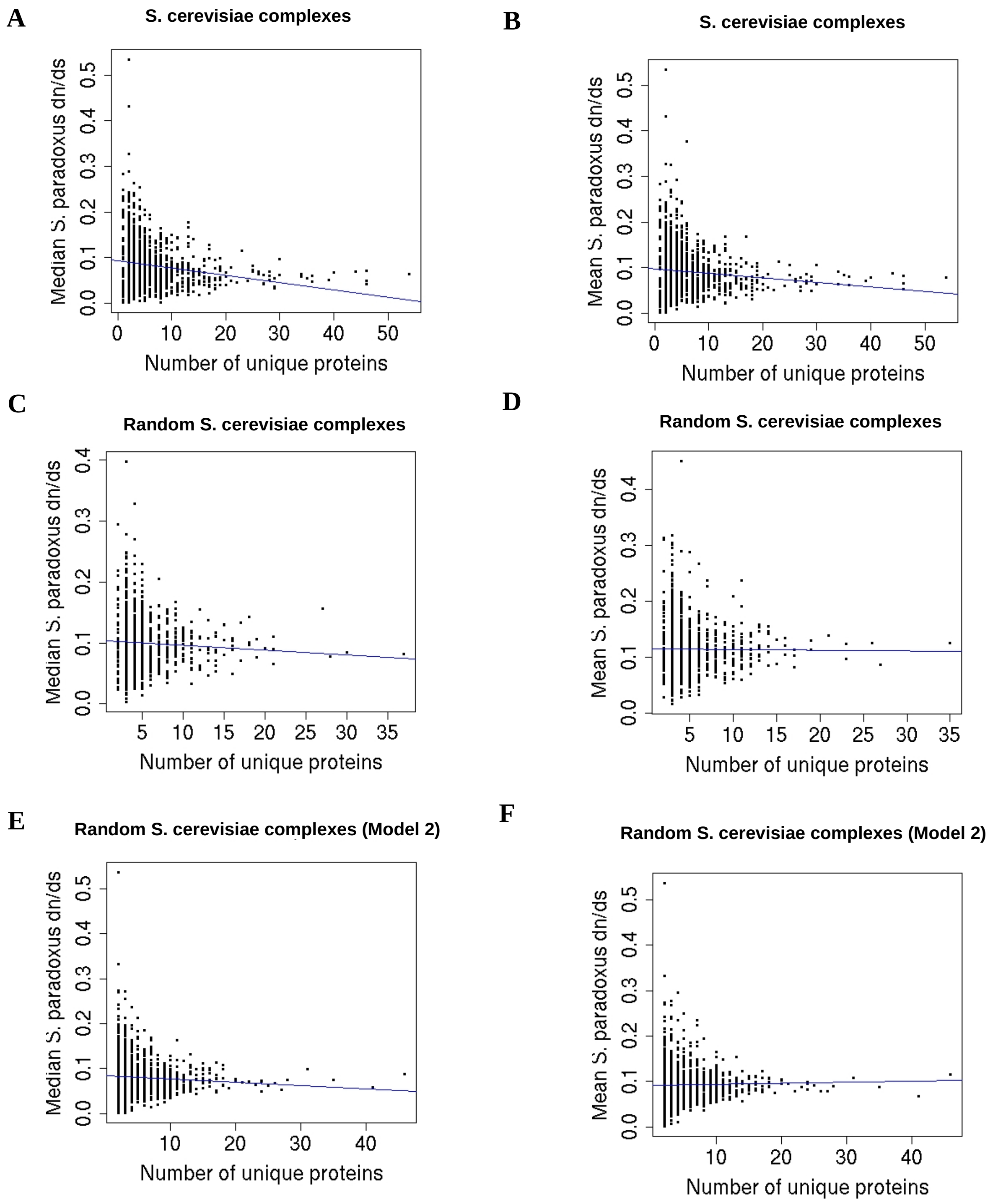

Figure S13c: Median dN/dS values from S. cerevisiae – S. paradoxus orthologs tend to decrease with increasing complex complexity for A) annotated complexes (t-test: $P < 3.5 \times 10^{-12}$), C) Model 1 random complexes (t-test: $P < 0.05$), and E) Model 2 random complexes (t-test: $P < 0.02$). However, such trend was only observed for B) annotated complexes when mean dN/dS ratios were plotted against complex complexity (t-test: $P < 1.5 \times 10^{-5}$).

S13d – Analysis of Yeast Complex Complexity and protein length using the median

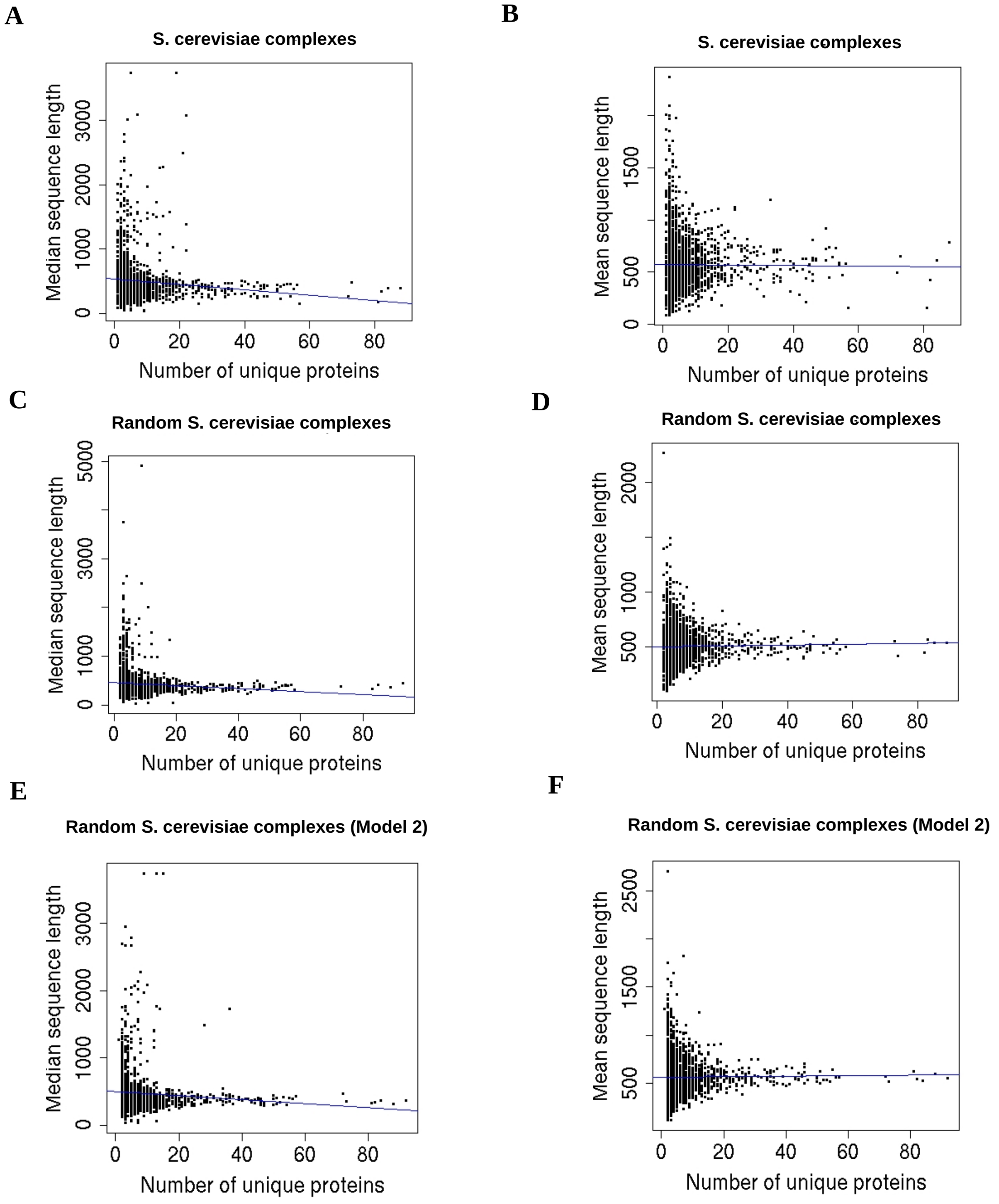

Figure S13d: Median sequence length tend to decrease with increasing complex complexity for A) annotated complexes (t-test: $P < 9 \times 10^{-7}$), C) Model 1 random complexes (t-test: $P < 2.2 \times 10^{-5}$), and E) Model 2 random complexes (t-test: $P < 5 \times 10^{-4}$). Note that for some model 2 random complex instances, P-values $< 2 \times 10^{-8}$ have been observed. Thus, like S13b when median lengths were examined, trends for random complex complexes have been observed to have lower P-values than trends associated with annotated complexes. However, such significant trends were not observed when mean sequence length is plotted against complex complexity.
