# Supplementary material for: An evolutionary and structural characterization of mammalian protein complex organization
Source: BMC Genomics. 2008 Dec 23;9:629. doi: 10.1186/1471-2164-9-629 (PMC2645396; doi:10.1186/1471-2164-9-629)
Supplement: Additional File 14 — Homogeneity of Protein Properties in Yeast Complexes. Deviation in pI, secondary structure and evolutionary rate between proteins in annotated yeast complexes are compared to those in random complexes. [file 1471-2164-9-629-S14.pdf]

Additional file 14: Homogeneity of Protein Properties in Yeast Complexes

Protein Complex Data	Property	Mean Stdev. Annotated	Mean Stdev. Random
S. cerevisiae	pI	1.6 ± 0.6 (1.7)	1.8 ± 0.5 (1.9)
S. cerevisiae	% Helix	15.9 ± 6.1 (16.0)	16.8 ± 5.8 (16.9)
S. cerevisiae	% Sheet	6.7 ± 2.9 (6.4)	7.1 ± 3.1 (6.8)
S. cerevisiae	% Coil	11.3 ± 4.9 (10.9)	12.5 ± 4.9 (12.3)
S. cerevisiae (against S. paradoxus)	*dN/dS*	0.06 ± 0.04 (0.06)	0.08 ± 0.13 (0.07)

Table S14: Homogeneity of Protein Properties in Yeast Complexes of 3 or more proteins

The standard deviations of protein property values for each annotated complex are significantly smaller than ones for complexes generated randomly by picking proteins with replacement (MC-test using 1000 complexes generated randomly with replacement: $P < 0.001$). The means ± standard deviation of these standard deviations for each property are shown in the last two columns along with median values (in parentheses).
