# Supplementary material for: An evolutionary and structural characterization of mammalian protein complex organization
Source: BMC Genomics. 2008 Dec 23;9:629. doi: 10.1186/1471-2164-9-629 (PMC2645396; doi:10.1186/1471-2164-9-629)
Supplement: Additional File 15 — Length difference of interacting proteins. Large proteins tend to interact with much smaller partners. [file 1471-2164-9-629-S15.pdf]

Additional file 15: Length difference of interacting proteins

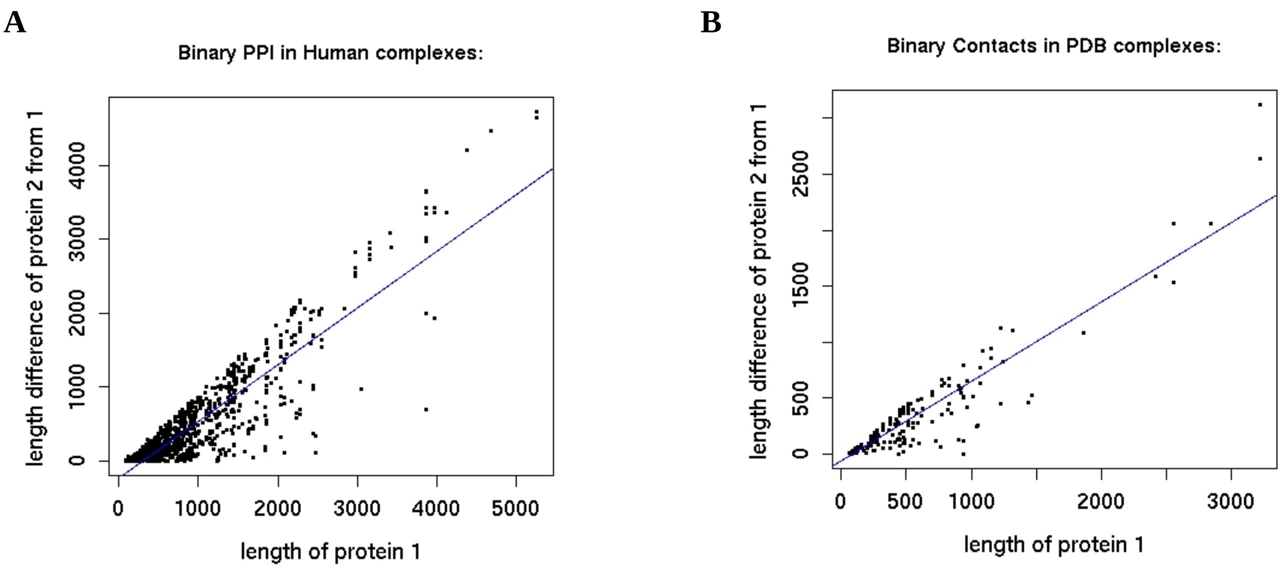

Figure S15. Large proteins tend to interact with much smaller partners
For each protein pair of different proteins, the length of the larger protein is plotted on the x-axis. The difference in length of its partner is plotted on y-axis. Proteins larger than the mean length on the x-axis tend to interact with relatively smaller proteins compared to proteins of smaller size. We see this trend amongst A) binary interactions within our complexes and B) contacting proteins in PDB structures of mammalian complexes (t-test: $P < 0.001$).

S15b – Length difference of interacting proteins (binned plots)

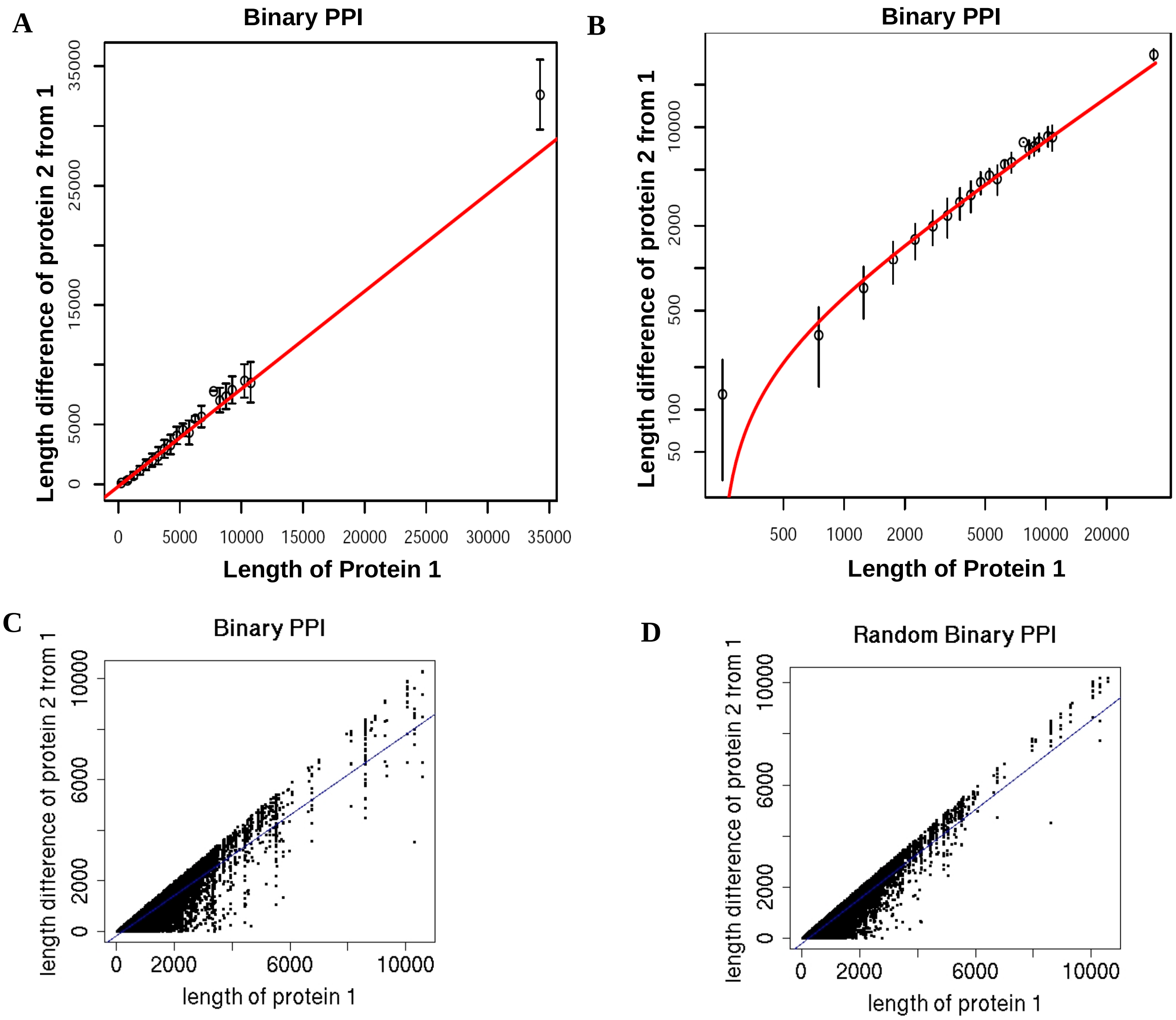

Figure S15b - Binned Plots. Figures S15bA and S15bB are derived from Figure 5A of the main text. The points from Figure 5A are placed into 500aa bins (circles representing the mean value at each bin and the bars indicate the first standard deviation). A log transformation was applied in Figure S15bB. In both S15bA and S15bB, larger proteins tend to have larger length differences with their interaction partners (t-test: $P < 0.001$).

Figure S15bC and S15bD are blown up replicas of Figure 5A and 5B without the proteins > 25000aa in length. The trends remain significant (t-test: $P< 0.001$). Even when proteins > 25000aa in length are included, points from Figure S15bC (5A) do not fit as well to a straight line as those from Figure S15bD (5B), perhaps due to historical, selective constraints or experimental error.
